# Direct wastewater extraction as a simple and effective method for SARS-CoV-2 surveillance and COVID-19 community-level monitoring

**DOI:** 10.1093/femsmc/xtad004

**Published:** 2023-01-12

**Authors:** Megan E J Lott, William A Norfolk, Cody A Dailey, Amelia M Foley, Carolina Melendez-Declet, Megan J Robertson, Stephen L Rathbun, Erin K Lipp

**Affiliations:** Department of Environmental Health Science, University of Georgia, 150 East Green Street, Athens, GA 30602, United States; Department of Environmental Health Science, University of Georgia, 150 East Green Street, Athens, GA 30602, United States; Department of Epidemiology and Biostatistics, University of Georgia, 101 Buck Road, Athens, GA 30606, United States; Department of Environmental Health Science, University of Georgia, 150 East Green Street, Athens, GA 30602, United States; Department of Environmental Health Science, University of Georgia, 150 East Green Street, Athens, GA 30602, United States; Department of Environmental Health Science, University of Georgia, 150 East Green Street, Athens, GA 30602, United States; Department of Epidemiology and Biostatistics, University of Georgia, 101 Buck Road, Athens, GA 30606, United States; Department of Environmental Health Science, University of Georgia, 150 East Green Street, Athens, GA 30602, United States

**Keywords:** wastewater surveillance, SARS-CoV-2, direct extraction, RT-qPCR, COVID-19, wastewater-based epidemiology

## Abstract

Wastewater surveillance has proven to be an effective tool to monitor the transmission and emergence of infectious agents at a community scale. Workflows for wastewater surveillance generally rely on concentration steps to increase the probability of detection of low-abundance targets, but preconcentration can substantially increase the time and cost of analyses while also introducing additional loss of target during processing. To address some of these issues, we conducted a longitudinal study implementing a simplified workflow for SARS-CoV-2 detection from wastewater, using a direct column-based extraction approach. Composite influent wastewater samples were collected weekly for 1 year between June 2020 and June 2021 in Athens-Clarke County, Georgia, USA. Bypassing any concentration step, low volumes (280 µl) of influent wastewater were extracted using a commercial kit, and immediately analyzed by RT-qPCR for the SARS-CoV-2 N1 and N2 gene targets. SARS-CoV-2 viral RNA was detected in 76% (193/254) of influent samples, and the recovery of the surrogate bovine coronavirus was 42% (IQR: 28%, 59%). N1 and N2 assay positivity, viral concentration, and flow-adjusted daily viral load correlated significantly with per-capita case reports of COVID-19 at the county-level (ρ = 0.69–0.82). To compensate for the method’s high limit of detection (approximately 10^6^–10^7^ copies l^−1^ in wastewater), we extracted multiple small-volume replicates of each wastewater sample. With this approach, we detected as few as five cases of COVID-19 per 100 000 individuals. These results indicate that a direct-extraction-based workflow for SARS-CoV-2 wastewater surveillance can provide informative and actionable results.

## Introduction

The coronavirus disease 2019 (COVID-19) is an infectious respiratory disease caused by the novel severe acute respiratory syndrome coronavirus 2 (SARS-CoV-2). Individuals infected with SARS-CoV-2 shed viruses and viral RNA through respiratory fluids, saliva, urine, and stool (Kitajima et al. 2020, Park et al. 2020, Tang et al. 2020, Wu et al. 2020). These excreta, collected by the sewage network, can be monitored in downstream wastewaters as a proxy for disease surveillance (Medema et al. 2020, Polo et al. 2020). The relationship between SARS-CoV-2 viral levels in wastewater and reported COVID-19 cases has been well-documented (Polo et al. 2020, Sanjuán and Domingo-Calap 2021, Wu et al. 2021). Previous studies have demonstrated that viral levels in wastewater correlate with COVID-19 incidence and hospitalization rates (Peccia et al. 2020, Galani et al. 2022). Wastewater surveillance is useful for detection of both asymptomatic and symptomatic cases of infection (Schmitz et al. 2021), and may serve as an early warning system for disease outbreaks or surges in transmission (Xagoraraki and O’Brien 2020, Ahmed et al. 2021, Schmitz et al. 2021). In these ways, wastewater surveillance provides an indiscriminate, noninvasive, and cost-effective approach to community surveillance for both COVID-19 and other diseases.

As an early response to the COVID-19 pandemic, wastewater surveillance for SARS-CoV-2 was implemented rapidly by research laboratories across the globe (Bivins et al. 2020). With the success of these initial efforts, wastewater surveillance is now being formally adopted into state-wide and national public health programs. A growing number of public health, wastewater utility, commercial, and other laboratories are expanding their capacity to conduct routine wastewater surveillance (Naughton et al. 2023). However, small and rural communities are often underrepresented in wastewater surveillance programs, due in part to local resource and infrastructure constraints (D’Aoust et al. 2021, Medina et al. 2022). To facilitate the adoption and implementation of wastewater surveillance more broadly, it is essential to develop and evaluate flexible workflows that can be readily adopted across a range of settings, community needs, and resource availability (D’Aoust et al. 2021, Keshaviah et al. 2021, McClary-Gutierrez et al. 2021).

Among the numerous workflows described for wastewater surveillance, primary concentration has been considered a critical step in the recovery and detection of SARS-CoV-2 viral RNA from wastewater (Lu et al. 2020, Juel et al. 2021). However, concentration–extraction methods have notable limitations. Membrane filtration, ultrafiltration, and ultracentrifugation are subject to highly variable recovery rates and target loss through multiple processing steps and the coconcentration of inhibitors (LaTurner et al. 2021, Pecson et al. 2021). Moreover, these methods can be both time consuming and costly (LaTurner et al. 2021). Marked by high start-up costs, specialized consumables, and long processing times, these concentration methods may present a barrier to the adoption of long-term, sustainable, and high-throughput workflows for wastewater surveillance.

In place of concentration–extraction approaches, direct extraction presents a rapid, high-throughput, and cost-effective option for recovery of SARS-CoV-2 from wastewater. With fewer processing steps, viral recovery by direct extraction is less variable, and often more efficient, recovering up to 6-fold more SARS-CoV-2 viral RNA than other concentration–extraction methods (LaTurner et al. 2021, Pecson et al. 2021, Whitney et al. 2021). Notably, the “Sewage, Salt, Silica, and SARS-CoV–2 (4S)” method for direct column-based extraction, developed by Whitney et al. (2021), has been adopted for several longitudinal wastewater surveillance studies (Acosta et al. 2021, 2022). In this method, 40 ml of influent wastewater are extracted using direct column-based extraction, utilizing common laboratory reagents. Results from this workflow have been significantly correlated with clinical cases COVID-19, indicating the reliability of direct-extraction approaches for wastewater surveillance of SARS-CoV-2 (Acosta et al. 2021, 2022, Greenwald et al. 2021, Whitney et al. 2021).

To further explore the utility of direct-extraction methods for wastewater surveillance of SARS-CoV-2, we developed a further simplified workflow: extracting SARS-CoV-2 viral RNA directly from small unconcentrated volumes (280 µl) of unamended influent wastewater. We applied this simplified workflow in a year-long longitudinal study of wastewater in Athens-Clarke County, Georgia (USA) to assess its application in informing trends in clinical case reports of COVID-19.

## Methods

### Setting

**Figure 1.**
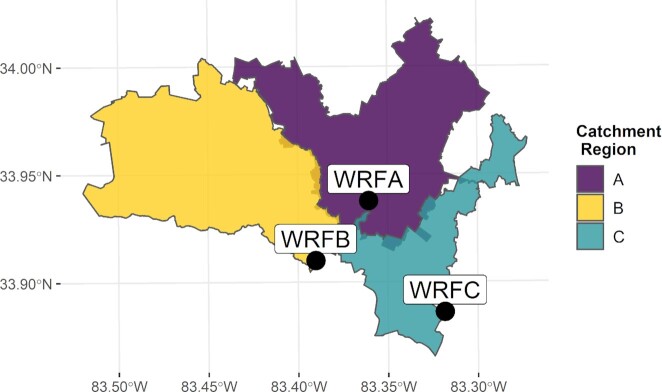
Athens-Clarke County, Georgia, USA, is served by three WRFs. WRF A (83.36°W, 33.93°N) serves a catchment area of approximately occupied by approximately 56 500 residents. WRF B (83.39°W, 33.91°N) serves a catchment area occupied by approximately 49 000 residents, and WRF C (83.31°W, 33.88°N) serves a catchment area occupied by approximately 29 000 residents (Athens-Clarke County Public Utilities Department 2020).

We implemented a simplified workflow for wastewater surveillance in Athens-Clarke County, Georgia, a county-township located in southeastern USA (Figure S1, Supporting Information). The three wastewater reclamation facilities (WRFs) in Athens-Clarke County service approximately 131 000 residents (Fig. 1) (Athens-Clarke County Public Utilities Department 2020).

### Sample collection and processing

Time-composite (24 h) wastewater influent samples were collected between 30 June 2020 and 30 June 2021 from each of the three WRFs in Athens-Clarke County. For each collection period, plant operators provided the total influent flow at each plant during the 24 h collection period as well as total suspended solids (TSS) levels. A 500 ml volume of the composite sample was collected in sterile polypropylene bottles and held on ice until processing. Samples were processed immediately upon receipt at the laboratory.

### Process control

Process controls were prepared by spiking bovine coronavirus (BCoV), carried in CalfGuard (Zoetis, Parsippany-Troy Hills, New Jersey, USA) into one subsample of influent wastewater from each WRF, to a final concentration of approximately 1.0 × 10^4^ gene copies ml^−1^ BCoV. The process controls were mixed end-over-end for 30 min at 4°C before viral RNA extraction. The original concentration of BCoV in CalfGuard was determined by RNA extraction and RT-qPCR in parallel with process controls.

### Viral RNA extraction

Viral RNA was extracted directly from small volumes (280 µl) of unconcentrated wastewater samples, process control samples, and extraction blanks [molecular-grade water (Corning, Corning, New York, USA)] using the QiAmp Viral RNA Mini Kit (catalog number 52906, Qiagen, Hilden, Germany) according to the manufacturer’s protocol. Up to six replicates of each sample were extracted (Figure S2, Supporting Information). Viral RNA was eluted from the spin columns in a final volume of 60 µl in Qiagen Buffer AVE. RNA extracts were stored at 4°C and analyzed by RT-qPCR within 24 h of extraction. Extracts were held at −20°C for long-term storage.

### Reverse-transcription real-time PCR (RT-qPCR)

A two-step RT-qPCR protocol was used for the detection of the SARS-CoV-2 N1 and N2 genes (U.S. Centers for Disease Control and Prevention (CDC) 2020) and the BCoV M gene (Decaro et al. 2008) (Table S1, Supporting Information). Viral RNA from each extraction replicate was converted to cDNA using an adapted protocol for Invitrogen M-MLV Reverse Transcriptase (catalog number 28025021, Thermo Fisher Scientific, Waltham, MA, USA). Briefly, sample RNA (3 µl), random hexamer (2.5 µM final concentration), dNTP mix (0.5 mM final concentration), and molecular-grade water were combined and heated to 65°C for 5 min. Following this incubation, M-MLV buffer (1 U), DTT (0.01 M), Superase RNAse Inhibitor (0.4 U), and M-MLV Reverse Transcriptase (1 U) were added to the solution, to a final reaction volume of 25 µl. The reaction was then incubated under the following conditions: 25°C for 10 min, 37°C for 50 min, and 70°C for 15 min.

Once converted to cDNA, each extraction replicate was assayed in triplicate by real-time quantitative PCR (qPCR) using TaqMan chemistry. The master mix for each assay was prepared according to the specifications in Table S2 (Supporting Information). All three targets were amplified under the following reaction conditions: 95°C for 2 min, followed by 40 cycles of 95°C for 3 s, and 55°C for 30 s (Step-One Realtime PCR system). Cq values were automatically generated by the Step-One Realtime PCR system.

### Standard curves

A SARS-CoV-2 plasmid standard (2019-nCoV_N_Positive Control, 4.12 kbp, catalog number 10006625, Integrated DNA Technologies, Coralville, Iowa, USA), including both the N1 and N2 genes, was linearized by enzymatic digestion with ScaI-HF (New England Biolabs, catalog number R3122S). Briefly, 10 µl of the plasmid was incubated with 5 U of ScaI-HF in a 50 µl reaction at 37°C for 60 min. The enzyme was inactivated at 80°C for 20 min. The BCoV standard was synthesized as a DNA Ultramer (Table S3, Supporting Information; Integrated DNA Technologies). Standards for SARS-CoV-2 and BCoV were assayed in triplicate to generate a standard curve for the three targets (Figure S3, Supporting Information).

### Inhibition control

To assess inhibition in downstream reactions, sample RNA (1.5 µl) was combined with 1.5 µl of an RNA Ultramer standard for BCoV (Table S3, Supporting Information; Integrated DNA Technologies). The BCoV RNA Ultramer (1.5 µl) was assessed in parallel in PCR-grade water (1.5 µl).

### SARS-CoV-2 RNA positive control

Synthetic RNA fragments of SARS-CoV-2 carrying the N1 and N2 targets (catalog number EURM019, MilliporeSigma, Burlington, MA, USA) were assayed in parallel with every batch of samples to account for run-to-run variability not related to sample matrix or extraction efficiency. From the standard, 3 µl (approximately 2.19 × 10^8^ copies) was reversed transcribed and amplified through the qPCR for N1 and N2 gene targets as described above.

### COVID-19 case data

County-level reports of PCR-confirmed cases of COVID-19 for Athens-Clarke County were accessed through the Georgia Department of Public Health (dph.georgia.gov). Data were provided by the date of PCR test result (Supplementary data). Cases were geocoded to each WRF catchment region by household address by the Georgia Department of Public Health in response to a request through the Public Health Information Portal (PHIP) (Request #: 55153, Supplementary data).

### Reporting quality

The Environmental Microbiology Minimum Information (EMMI) Guidelines from Borchardt et al. (2021) are addressed in Table S4 (Supporting Information).

## Data analysis

All data wrangling and statistical analyses were performed using R (v.4.0.4) in RStudio v.1.1.453. Analysis scripts are available on Github (https://github.com/lipplab-uga/wbe_manuscript_2022). Wastewater surveillance data for Athens-Clarke County has been reported in real-time on a public dashboard, which can be accessed through Github (https://github.com/lipplab-uga/covid_wastewater_lipplab_athens). Summary statistics for recovery, frequency of target detection, Cq values, concentrations, and viral loads were assessed and visualized using the “ggstatsplot” package v.0.9.3 (Patil 2021). Additional statistical details are included in Text S1 (Supporting Information).

### Inhibition

A sample was considered inhibited if the change in Cq between the sample and inhibition control was greater than 3.33 cycles. This threshold was determined by examining the median and interquartile deviation of all Cq differences calculated (Figure S4, Supporting Information). Inhibition was noted (Figure S5, Supporting Information), but not corrected in downstream data analysis.

### Recovery

Viral recovery was calculated by estimating the input concentration of BCoV (cp [copies] µl^−1^) and recovered copies. BCoV recovery was reported as a %, according to Equation (1).

(1) \begin{eqnarray*}
Recovery\ \left( \% \right)\ = \frac{{Copies\ Recovered}}{{Copies\ Spiked}} \cdot 100.
\end{eqnarray*}

### Limits of detection and quantification

We determined the limit of detection (LoD) and limit of quantification (LoQ) by referring to the most basic definitions of these measures as provided by the Clinical and Laboratory Standards Institute, where the LoD is defined as the smallest amount of analyte that can be reliably detected by the method, and the LoQ is defined as the smallest amount of analyte can reliably quantified by the method (The Clinical and Laboratory Standards Institute 2012). For RT-qPCR, the Cq results are usually normally distributed (Forootan et al. 2017); thus, we defined the LoQ and detection based on the deviations of observed Cq values from the expected normal distribution. When Cq values were plotted against a normal quantile–quantile plot, tails at the upper quantiles were considered artifacts of the assay’s detection limits. The lower inflection point of the tail was defined as the LoQ; beyond this Cq value, we could not reliably quantify the target analyte using the standard curve. The upper inflection point of the tail was defined at the LoD; Cq values beyond this inflection point could not be reliably detected or distinguished from noise.

### Reaction concentration

Concentrations of SARS-CoV-2 were first determined as copies per µl (cp µl^−1^) per RT-qPCR reaction (Supplementary data). For reactions with a Cq below the LoQ, the concentration of the gene target was determined using the associated standard curve. For reactions which amplified between the LoQ and LoD, the reaction was assigned a concentration equivalent to half of the LOQ. For reactions which amplified after the LoD, or did not amplify within 40 cycles, the reaction was assigned a concentration equivalent to half of the LoD. The geometric means of the triplicate RT-qPCR reactions were calculated and reported as ${C}_{RXN}\ $ in cp µl^−1^.

### Sample concentration

The concentration of the original wastewater sample (${C}_{WW}$) was estimated from the reaction concentration ${C}_{RXN}\ $ for each extraction replicate according to Equation (2).

(2) \begin{eqnarray*}
{C}_{WW} = {C}_{RXN} \cdot \left( {\frac{{{V}_{qPCR}}}{{{V}_{cDNA}}}{\mathrm{\ }}} \right) \cdot \left( {\frac{{{V}_{RT}}}{{{V}_{RNA}}}} \right) \cdot \left( {\frac{{{V}_{Elution}}}{{{V}_{WW}}}} \right) \cdot \frac{{1.0\ \times\ {{10}}^6\ uL}}{{1\ L}}.
\end{eqnarray*}

Equation (2) accounts for the initial wastewater extraction volume, ${V}_{WW}$ (280 µl), the elution volume, ${V}_{Elution}$ (60 µl), the volume of template (sample) RNA extract into the reverse-transcription reaction, ${V}_{RNA}$ (3 µl), the volume of the RT reaction, ${V}_{RT}$ (25 µl), the volume of the template cDNA, ${V}_{cDNA}$ (2 µl), and the volume of the qPCR reaction ${V}_{qPCR}$ (20 µl). The ${C}_{WW}\ $ for each influent sample was then reported as the geometric mean across all extract replicates of that sample, in copies per liter (cp l^−1^).

### Viral load

To account for potential dilution due to inflow and infiltration and to normalize viral levels across facilities, viral concentrations for each influent sample were converted to total daily viral load (*L_WW_*) as copies per day (cp day^−1^) based on Equation (3).

(3) \begin{eqnarray*}
{L}_{WW}\ = {\mathrm{\ }}{C}_{WW}{\mathrm{\ }} \cdot \ {V}_{Influent}.
\end{eqnarray*}

In Equation (3), ${V}_{Influent}$ is the total volume of influent wastewater (in liters) received by the WRF during the 24 h that comprised the sample collection period. Viral load was examined per target (N1 or N2) as well as the geometric mean of the N1 and N2 viral loads. For each collection period, the total viral load was estimated for each WRF and for the county as a whole. The county-wide viral load was estimated as the sum of the viral load from each WRF.

### Recovery-adjusted viral load

Viral loads were adjusted to account for viral recovery by correcting the viral concentration by the % of BCoV recovered from the process control. The recovery-adjusted viral load (${R}_{WW}{\mathrm{\ }}$) was calculated according to Equation (4).

(4) \begin{eqnarray*}
{R}_{WW}\ = {\mathrm{\ }}\frac{{{C}_{WW}}}{{Recovery}}{\mathrm{\ }} \cdot \ {V}_{Influent}.
\end{eqnarray*}

### Comparison of wastewater SARS-CoV-2 detection to clinical cases

For all statistical comparisons, clinical case reports of COVID-19 were evaluated as per-capita values (per 100 000) of the 7-day moving average (7-dma) of RT-qPCR positive tests, by report date. Spearman’s correlations between measures of SARS-CoV-2 in wastewater (assay positivity, concentration, viral load, and recovery-adjusted viral load) were assessed for both county-level and catchment-level measures of SARS-CoV-2 and geocoded cases of COVID-19.

Lead and lag times were characterized for the full time series by assessing the correlation between county-level wastewater measures of SARS-CoV-2 and the county-level reported cases for all dates 6 days prior (−6:0) and 6 days following (0:6) the wastewater collection period. In this assessment, wastewater viral loads “lag” clinical case reports if correlations were strongest with clinical cases reported prior the wastewater collection period (−6:0). Wastewater viral loads “lead” clinical case reports if correlations were strongest with clinical cases reported following the wastewater collection period (0:6).

The case LoD, $Cas{e}_{LoD},$ was estimated as the minimum number of reported cases that would result in a positive detection of SARS-CoV-2 in wastewater. A simple linear regression was performed between the log10-transformed incidence of reported cases (per-capita, 7-dma) and the total SARS-CoV-2 wastewater assay positivity. The slope (*m*) and y-intercept (*b*) of the regression line were used to determine the ${C}_{LoD}.$

## Results

In total, 254 time-composite samples were collected over the year-long study period, including 85 from WRF A, 85 from WRF B, and 84 from WRF C (Table 1). Total daily flows ranged from 4.96 million liters per day (Ml day^−1^) to 39.10 Ml day^−1^, with highest flow at WRF A (mean 20.51 Ml day^−1^) and lowest at WRF C (mean 6.83 Ml day^−1^) (Supplementary data). TSS ranged from 60 to 1220 mg l^−1^ with a mean of 281 mg l^−1^ (Supplementary data).

**Table 1.** Samples collected from WRFs in Athens-Clarke County, and their associated characteristics.

WRF	Number of samples	Population served^a^	Capacity (Ml/day)	Daily flow (Ml/day +/− SD)^b^	TSS (mg/l +/−SD) ^b^
A	85	56 500	53	20.51 (3.58)	275 (123)
B	85	49 000	38	13.98 (3.01)	261 (141)
C	84	25 500	15	6.83 (1.29)	307 (207)

a. Population estimates referenced from the Athens-Clarke County Public Utilities Department’s 2020 Service Delivery Plan Update.

b. Estimated from collection periods included in this study.

### Recovery

Viral recovery was estimated from direct extraction and RT-qPCR detection using the 165 process control samples seeded with BCoV (Supplementary data). The median recovery of BCoV was ∼ 42% (IQR: 28%, 59%) and ranged from 0.1% to 260% (Figure S7A, Supporting Information). Recovery of BCoV from samples collected at WRF A was significantly higher than those collected at WRF C (*P* = .02) but was similar to those collected at WRF B (*P*= .25, Figure S7B, Supporting Information). Recovery did not differ between samples collected from WRF B and WRF C (*P*= .25). Recovery of BCoV did not correlate with TSS of the influent sample, nor with the total influent flow of the sample collection period (Figure S7C and D, Supporting Information).

### Detection of SARS-CoV-2 N1 and N2 gene targets

Using the direct extraction assay, SARS-CoV-2 viral RNA (N1 or N2 gene target) was detected in 193 of the 254 (76%) composite influent wastewater samples (Table 2). The N1 gene target was detected in 159/254 influent samples (63%) and 151/248 influent samples were positive for the N2 gene target (61%). Just under half of the influent samples (117/248) were positive for both targets (47%). SARS-CoV-2 was not detected by either target in 59/248 influent samples (24%). Only 38/248 influent samples (15%) were positive for N1, but not N2. Comparably, 34/248 influent samples (14%) were positive for N2, but not N1. Detection frequency was not significantly different between gene targets (*P*= .69), nor was detection frequency significantly different between WRFs (*P*= .11).

**Table 2.** Frequency of SARS-CoV-2 detection, aggregated by WRF and gene target.

WRF	Gene target	Samples positive (%)
A	N1	53/85 (62%)
	N2	51/83 (61%)
	N1 or N2	64/85 (75%)
	N1 & N2	40/83 (48%)
B	N1	55/85 (65%)
	N2	58/83 (70%)
	N1 or N2	68/85 (80%)
	N1 & N2	45/83 (54%)
C	N1	51/84 (61%)
	N2	42/82 (51%)
	N1 or N2	61/84 (73%)
	N1 & N2	32/82 (39%)
All Facilities	N1	159/254 (63%)
	N2	151/248 (61%)
	N1 or N2	193/254 (76%)
	N1 & N2	117/248 (47%)

### Limits of detection and quantification

Observed Cq values for the N1 assay ranged from 32.16 to 39.02 and observed Cq values for the N2 assay ranged from 31.23 to 39.03 (Figure S6, Supporting Information). There was no significant difference in observed Cq values between the two targets (*P* = .44). The LoQ for the N1 assay was determined to be Cq 36.96, equivalent to 3.66 × 10^6^ cp l^−1^ in wastewater. The LoD of the N1 assay was Cq 37.14 (3.22 × 10^6^ cp l^−1^ in wastewater). The LoQ and LoD for the N2 assay were Cq 37.00 (2.47 × 10^7^ cp l^−1^ in wastewater) and Cq 37.10 (2.18 × 10^7^ cp l^−1^ in wastewater), respectively. The number of RT-qPCR assay replicates and extraction replicates above, below, and between the LoD and LoQ are summarized in Table S5 (Supporting Information).

### Viral concentration

Concentrations of SARS-CoV-2 in influent samples ranged from below the LoD to 2.12 × 10^7^ cp l^−1^, based on the N1 target, and from below the LoD to 5.99 × 10^7^ cp l^−1^ based on the N2 target (Figure S8A, Supporting Information). Concentrations of N2 were significantly greater than N1 (*P*< .001), but concentrations were significantly correlated between the two assays (ρ *=*0.59,*P*< .001; Figure S8B, Supporting Information). Concentrations of SARS-CoV-2 did not differ significantly between the three WRFs (*N1: P*= .64,*N2: P*= .11; Figure S10A, Supporting Information).

### Daily viral load

When adjusted for influent flow volume, daily viral loads of influent samples ranged from 9.16 × 10^12^ to 1.97 × 10^14^ cp day^−1^ by the N1 target (Figure S9A, Supporting Information). Estimates of viral load by the N2 gene target ranged from 6.23 × 10^13^ to 9.37 × 10^14^ cp day^−1^ and were significantly greater than estimates by the N1 target (*P*< .001; Figure S9B, Supporting Information). Viral loads estimated by the N1 target were significantly correlated to estimates by the N2 target (Figure S9B, Supporting Information). Viral load differed significantly between WRFs (*P*< .001), with viral loads highest at WRF A, followed by WRF B, and WRF C, generally following the influent volumes of each plant (Figure S10B, Supporting Information).

### Correlation with COVID-19 cases

**Figure 2.**
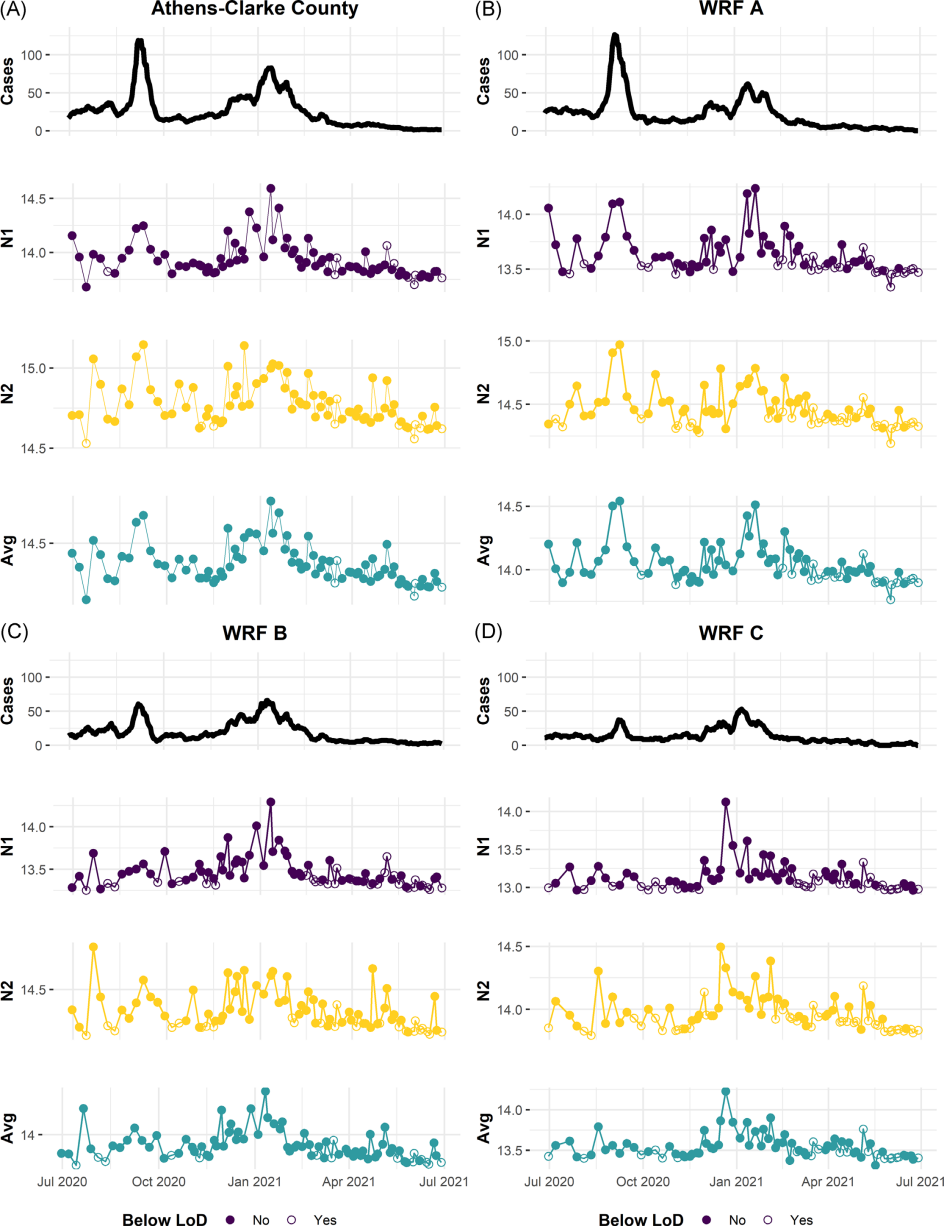
Wastewater viral loads for SARS-CoV-2 and reported COVID-19 cases in Athens-Clarke County (30 June 2020, and 30 June 2021). Results are reported at the county **(A)**, and Water Reclamation Facility (WRF) catchment scales **(B)–(D)**. Reported cases are presented as a 7-dma, per 100 000 individuals. SARS-CoV-2 total viral load was monitored through a direct-extraction approach and quantified using the N1 and N2 gene targets, and the geometric mean (Avg) of the two measures. Samples with no detectable levels of SARS-CoV-2 (below the LoD, < LoD), are annotated with open circles.

**Figure 3.**
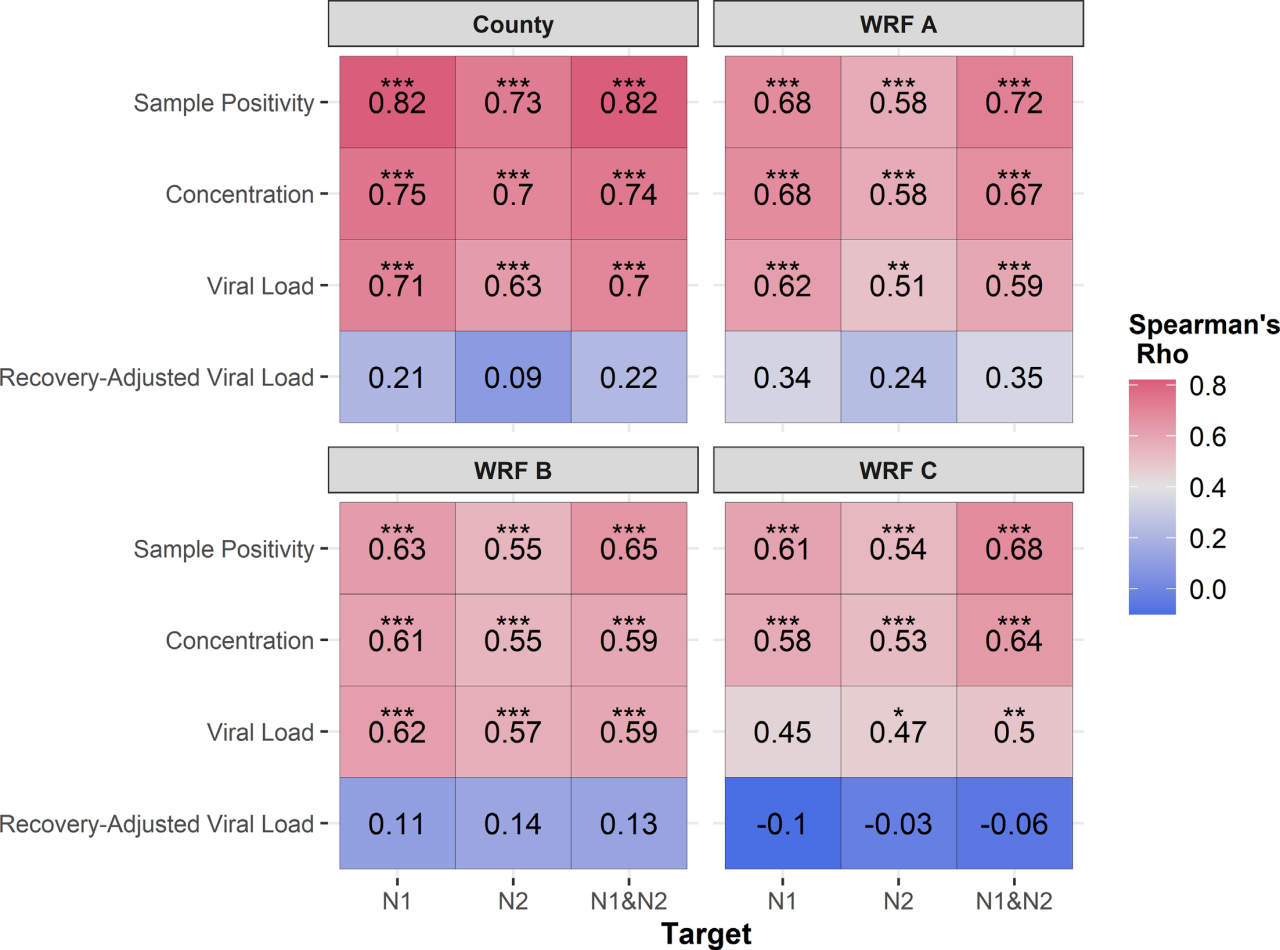
Correlations between measures of SARS-CoV-2 in wastewater (sample positivity, concentration, daily viral load, and recovery-adjusted viral load) and reported cases of COVID-19 (per-capita, 7dma). Assay positivity of SARS-CoV-2 was examined separately for each assay target (N1 or N2), and as the sum of all assays conducted (N1 and N2). Concentration, daily viral load, and recovery-adjusted viral load were examined separately for each assay target (N1 or N2), and as the geometric mean of N1 and N2 assays (N1 and N2). Correlations were examined at the county-level (all WRFs) and at the level of each WRF catchment, without adjusting for lead or lag times. Spearman’s rho is reported with significance level (*0.05, **0.001, and ***0.001).

County-level viral loads in wastewater peaked in early September 2020 and again in January 2021 (Fig. 2A). These peaks corresponded with county-level surges in COVID-19 cases (Fig. 2A). Correlations between viral loads (N1, N2, and the geometric mean of N1 and N2) and reported cases were strongest when observed at the county-level (ρ = 0.69–0.73, Fig. 3). At the catchment level, total viral loads at WRF A and WRF B increased contemporaneously with geocoded case reports (per-capita, 7dma), peaking in early September 2020 and again in mid-January 2021 (Fig. 2B and C). For these catchments, viral loads were significantly correlated with case reports (ρ = 0.59–0.62; Fig. 3). Correlations were weakest at WRF C (ρ = 0.45–0.50, Fig. 3). In this small catchment, viral loads fluctuated sporadically as reported cases increased in September 2020; a clear peak in viral load was not observed until the surge in cases in January 2021 (Fig. 2D).

Across all scales, assay positivity was more strongly correlated with reported cases than either SARS-CoV-2 concentration or flow-normalized daily viral load (ρ = 0.54–0.82, Fig. 3). Assay positivity was most strongly correlated with reported cases when observed at the county-level and when examining positivity across both assays combined (N1 and N2, ρ = 0.82, Fig. 3). Correcting the total viral load for BCoV recovery significantly reduced correlations across all scales (ρ = −0.06 to 0.35, Fig. 3).

### Estimated lead and/or lag time between wastewater detection and case reports

When examined at the county-level, daily viral loads (N1, N2, and geometric mean) and total assay positivity were all significantly correlated with the number of cases reported each day prior and each day following wastewater collection, over the span of the analysis (−6 to 6 days; Figure S10, Supporting Information). Strongest correlations were typically observed with 3-day lead, though no definitive lead or lag time could be determined.

### Case LoD

**Figure 4.**
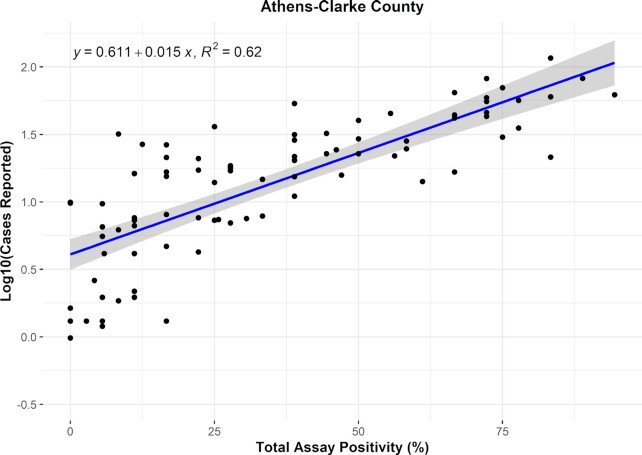
Estimated minimum number cases of COVID-19 to result in a detectable signal of SARS-CoV-2 in wastewater. The case LoD was determined by assessing the correlation between log10-transformed reported cases (per-capita, 7-dma), and total assay positivity. The linear regression between these values was then determined to estimate the minimum number of cases, *y*, required for a positive detection in *x*, a ratio of one positive assay out of the total numbers of assays conducted.

At the county-level, total assay positivity of all N1 and N2 assays for SARS-CoV-2 increased proportionately with an increase in the number of reported clinical cases (Fig. 4). Based on the equation of the regression line between reported clinical cases and wastewater assay positivity, we estimated the minimum number of cases of COVID-19 required to detect SARS-CoV-2 in wastewater in at least one assay (2.8% positivity). The 2.8% wastewater assay positivity value corresponds with 4.47 cases (95% CI 3.51–5.75) per 100 000 individuals. Based on the results of this study, we estimate the case LoD for wastewater surveillance to be approximately five cases per 100 000 individuals.

## Discussion

As SARS-CoV-2 wastewater surveillance initiatives transition into formally established state and nationwide programs, there is a growing need to develop and validate easily adoptable methods for testing that can be used in a variety of lab settings. In this study, we implemented a simplified approach for detection and quantification of SARS-CoV-2 in wastewater, using direct column-based RNA extraction without concentration or preprocessing. Across a year-long study period, we detected and quantified SARS-CoV-2 viral RNA from low-volumes of unconcentrated wastewater samples, establishing a robust time series of wastewater viral loads that correlated significantly with local reported cases of COVID-19. Without the need for expensive start-up equipment or specialized expertise, this approach can be easily adopted for sustainable, long-term surveillance programs from which results can be translated into public health action.

### Processing efficiency

The direct extraction method presented here requires limited start-up and only a few consumables (e.g. commercial extraction kits, microcentrifuge, and a quantification platform). Processing low volumes of influent wastewater also allows for rapid processing times and high throughput analysis of multiple samples. With this approach, sample processing, extraction, and analysis can be completed by a single technician within a 6-h period, including RT-qPCR run times (2 h for extraction, 1 h to prepare for RT-qPCR, and 2 h of run time for RT-qPCR). These turn-around times are comparable to reports by Whitney et al. (2021) for the “4S” direct extraction method, which requires approximately 2 h for the direct extraction of up to 40 ml of influent wastewater. In contrast, processing times for concentration–extraction methods can span between 30 min to 4.6 h for sample concentration alone (LaTurner et al. 2021).

Over the course of the study, we increased the number of direct column-based extractions to six replicates per influent sample. Increasing the number of extraction replicates was intended to both increase the representative sample volume analyzed and address the stochasticity in the distribution of viruses in wastewater. Increasing the number of extraction replicates did not significantly increase processing times but did increase the per-sample price of sample extraction and RT-qPCR. Per-sample costs may be limited by substituting a commercial extraction kit with kit-free consumables and reagents (Whitney et al. 2021).

### Recovery

We implemented this direct extraction approach in part to minimize the variability in viral recovery that has been previously attributed to concentration–extraction methods (LaTurner et al. 2021, Pecson et al. 2021). Based on the BCoV process control used in this study, the median viral recovery by direct extraction was approximately 42%, which is higher than that reported by LaTurner et al. (2021) for concentration–extraction methods with either HA filtration, PEG precipitation, or ultracentrifugation (0.96%, 0.08%, and 0.36%, respectively). To estimate recovery for direct extraction approaches, LaTurner et al. (2021) employed a similar method reported here, extracting BCoV from 300 µl of influent wastewater. However, LaTurner et al. (2021) reported the extraction efficiency of their direct extraction method to be 3.84%, a significant disparity which could be explained in part by minor methodological differences between groups (e.g. differences in sample preprocessing, quantification platform, or wastewater matrix).

The recovery of BCoV varied consistently with changes in primary CalfGuard stock concentration but was not significantly associated with changes in the composition of the sample matrix, including TSS or influent flow volume. Correcting wastewater viral load to account for BCoV recovery significantly reduced correlations with reported cases and likely introduced more variability than it resolved (Feng et al. 2021, Kantor et al. 2021). However, monitoring the recovery of the process control as a quality control measure is important in reporting results.

### Limits of detection and quantification

A notable limitation of direct extraction is the relatively high methodological limits of detection and quantification. Based on results from this study, we estimated the LoQ for this method to be 0.2 copies µl^−1^ per reaction, equivalent to 17 copies µl^−1^ per RNA template, or 3.66 × 10^6^ cp l^−1^ of wastewater, based on the N1 gene target. The LoQ for the N2 gene target was approximately 10-fold higher, 1.38 cp µl^−1^ per reaction, equivalent to 1.15 × 10^2^ cp µl^−1^ per RNA template, or 2.47 × 10^7^ cp l^−1^ of wastewater. Transitioning from two-step to one-step RT-qPCR may reduce the LoQ 10-fold by eliminating a dilution step from the RT to qPCR reactions. However, the LoQ reported here is 1000-fold higher than the LoQ for HA filtration, PEG precipitation, and ultracentrifugation reported by LaTurner et al. (2021), at 3.07 × 10^3^, 2.56 × 10^3^, and 7.67 × 10^3^ cp l^−1^, respectively. To compensate for the method’s high LoD, we increased the number of extraction replicates to six replicates per facility and sampling date, based on the maximum capacity of our microcentrifuge. Future studies should evaluate the number of extractions replicates required to optimize detection while minimizing extraction costs.

### N1 and N2 comparison

SARS-CoV-2 viral loads were monitored by both the N1 and N2 assays in parallel. While this approach increased the per-sample cost of analysis, it improved the likelihood of detection of SARS-CoV-2 during periods of low viral concentration. Notably, examining assay positivity of both targets improved correlations with reported cases of COVID-19. Analyzing samples for multiple targets may reduce the likelihood of false negatives, especially in the event when viral mutations of circulating variants diminish the efficiency of primer or probe binding (Wang et al. 2020).

### Wastewater surveillance and public health

In this study, we documented two distinct surges in SARS-CoV-2 viral loads in wastewater, in September 2020 and January 2021, which corresponded with two major peaks in reported COVID-19 cases in Athens-Clarke County. County-level SARS-CoV-2 viral loads, viral concentration, and assay positivity were significantly correlated with the 7-dma of per-capita reported cases on the date of sample collection (ρ = 0.69–0.82). The strength of these correlations is consistent with reports by Hoar (2022) who examined wastewater viral loads in New York City catchment regions serving between 120 000 and 1.2 million individuals. We found that associations between wastewater detection and reported cases were strongest when data were examined at larger scales (county-level), but the strength of the correlation decreased proportionately with the size of the catchment population, a finding also reported by others (Nagarkar et al. 2021, Acosta et al. 2022).

SARS-CoV-2 was detected in a majority (76%) of samples collected during this study. Only on five occasions, out of a total of 84 collections periods, did the viral concentration fall below the LoD for both gene targets, across all three influent samples. On these occasions, reported cases of COVID-19 fell between 1 and 10 cases per day, respectively (per-capita, 7dma). Based on the linear regression between reported cases and wastewater assay positivity, we estimated that SARS-CoV-2 viral RNA could be positively detected in wastewater when cases of COVID-19 exceed approximately five cases per day, per 100 000 individuals. This case LoD is comparable to those reported previously (Greenwald et al. 2021, Hoar et al. 2022, Wu et al. 2021). Greenwald et al. (2021) estimated the case LoD to be 2.4 cases per 100 000 individuals, the approximate case rate that would result in 95% RT-qPCR assay positivity. By examining the linear regression between reported cases and daily viral load, Hoar et al. (2022) determined the case LoD of wastewater surveillance to range from two to eight cases per day, per 100 000 individuals. Wu et al. (2021) estimated the case LoD by examining 1751 wastewater samples sourced from 159 US counties of varying case incidence. Using an exponential decay model between case incidence and sample positivity, Wu et al. (2021) estimated the case LoD to be approximately 13 cases per 100 000 individuals. While these estimates are comparable in magnitude, measures of the case LoD are not well-standardized. Quantitative methods to estimate the case LoD vary from study to study and warrant further investigation to account for differences in study design, sample processing, catchment scales, or the efficiency of local case reporting.

The approach for wastewater surveillance presented here is intended to complement clinical case reporting of COVID-19. While wastewater surveillance has been proposed as an early warning system, our results do not indicate a significant lead or lag time over case reporting. Instead, the primary value of this approach is in providing an independent estimate of disease trends, i.e. not subject to the same biases associated with clinical reporting (Olesen et al. 2021). In Athens-Clarke County, clinical case reporting tends to be most volatile during surges of COVID-19 cases. During these surges, the influx of positive clinical tests exceeds the normal operating capacity of the health district’s reporting workflow, resulting in atypical or delayed reporting times that can obfuscate true trends in disease prevalence (Hannah-Leigh Crawford, Epidemiologist Assistant, Georgia Department of Public Health, personal communication, 17 February 2022). It is during these surges of COVID-19 that we observe the most consistency in our wastewater surveillance data. Viral shedding from an increase of circulating cases results in consistent detection and more accurate quantification of SARS-CoV-2 from wastewater. In this way, wastewater surveillance data corroborates clinical case data, while providing an additional layer of data to better approximate the true temporal trend of a given outbreak. This additional layer of information becomes increasingly important in settings where clinical cases are widely underreported. As clinical testing rolls back, and at-home testing becomes widely adopted, wastewater surveillance data can independently confirm the presence of circulating cases and identify potential outbreaks that may not be reflected in clinical reports alone.

## Conclusion

Bypassing primary sample concentration, we detected SARS-CoV-2 in influent wastewater through direct extraction of small sample volumes. This method was appropriate for wastewater surveillance in our area of Athens, Georgia, serving a population of approximately 131 000 individuals, where the average number of reported cases was approximately 27 cases per 100 000 individuals across the study period, between June 2020 and June 2021. With our approach, we generated an informative time series of SARS-CoV-2 wastewater viral loads that correlated with reported cases of COVID-19 across catchment and county-level scales. To compensate for the method’s high LoD (approximately 10^6^–10^7^ copies l^−1^ in wastewater), we extracted multiple small-volume replicates of each wastewater sample. With this approach, we detected as few as five cases of COVID-19 per 100 000 individuals. This work demonstrates that direct extraction may be a simple and sustainable approach to wastewater surveillance, even during periods of low case incidence. As state and nation-wide surveillance programs expand, this workflow may be an attractive option for newly on-boarded water utilities or public health laboratories across a range of settings and resource availability.

## Supplementary Material

xtad004_Supplemental_Files
