# Supplementary material for: Direct wastewater extraction as a simple and effective method for SARS-CoV-2 surveillance and COVID-19 community-level monitoring
Source: FEMS Microbes. 2023 Jan 12;4:xtad004. doi: 10.1093/femsmc/xtad004 (PMC10117872; doi:10.1093/femsmc/xtad004)
Supplement: xtad004_Supplemental_Files [file xtad004_supplemental_files.zip › Lott_et_al_FEMS_2022_Supplemental_Tables_Figures_Revised.docx]

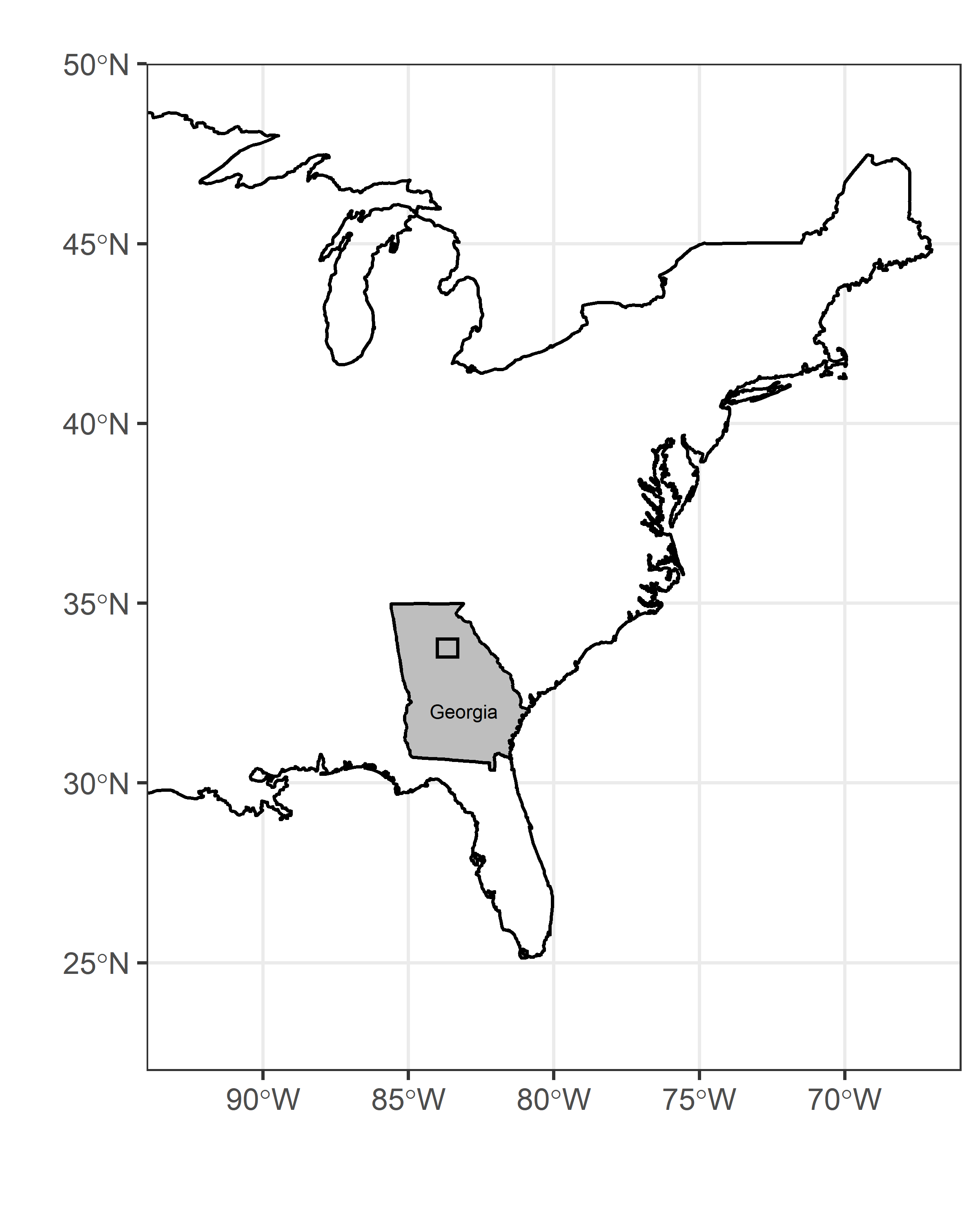


**Supplemental Figure 1.** Wastewater surveillance was conducted in Athens-Clarke County, Georgia located in southeastern United States. The study region is noted by a bounding box in the northeastern corner of the state.


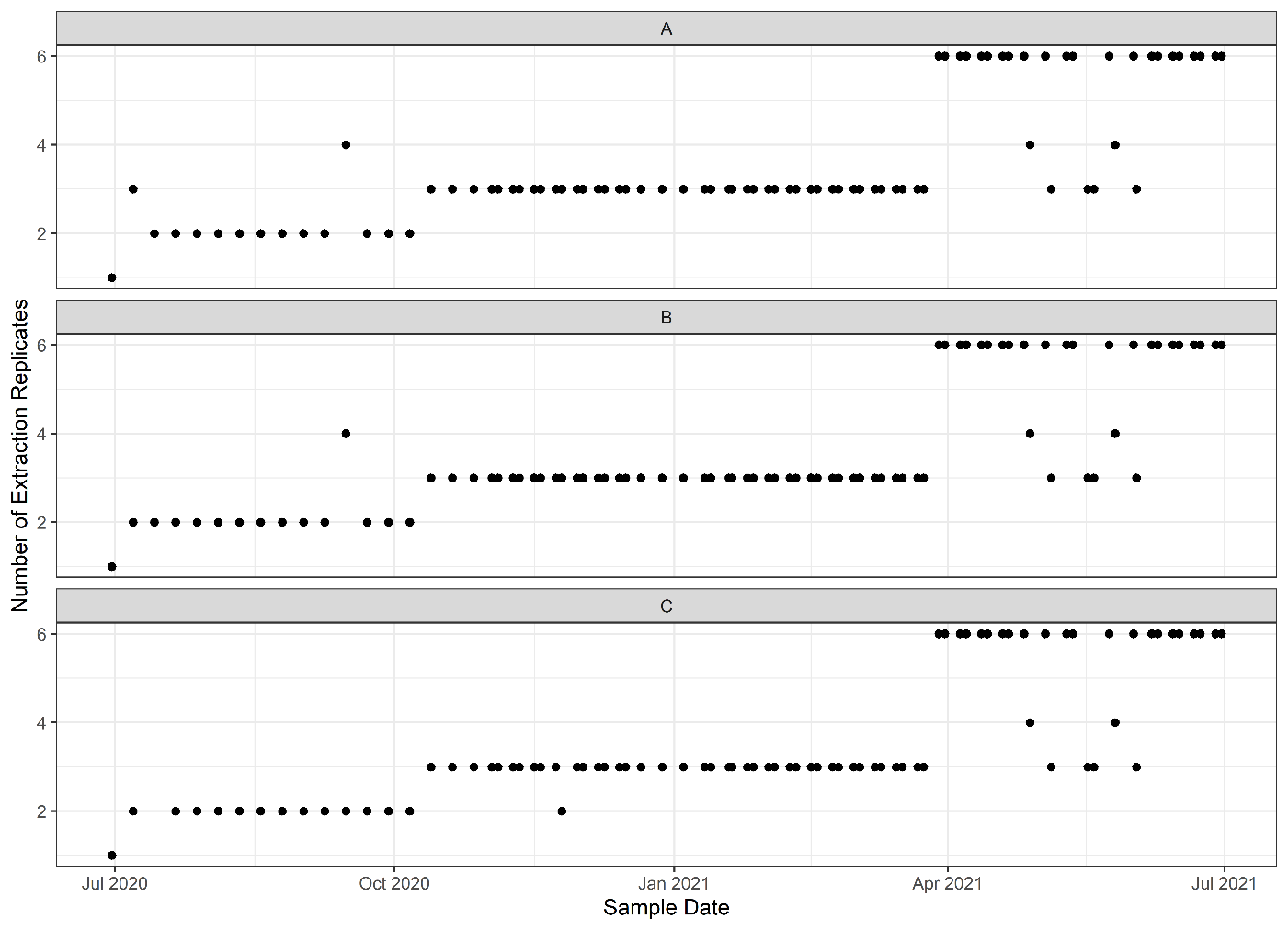

**Supplemental Figure 2.** Sampling effort for wastewater surveillance in Athens-Clarke County, GA, USA, between June 30, 2020 and June 30, 2021. Time-composite influent wastewater samples (24 h) were collected from three wastewater reclamation facilities (WRF A, WRF B, and WRF C). Samples were processed through direct column-based extraction, in replicates of one, two, three, four, or six.

**Supplemental Table 1.** Primers and probes used to target N1 and N2 genes specific to SARS-CoV-2 and the M gene specific to the bovine coronavirus process control.

| Gene Target | Sequence | Source |
| --- | --- | --- |
| N1,  SARS-CoV-2 | F: 5’-GAC CCC AAA ATC AGC GAA AT-3’ | U.S. Centers for Disease Control and Prevention (CDC) 2020 |
|  | R: 5’-TCT GGT TAC TGC CAG TTG AAT CTG-3’ |  |
|  | Probe: 5’-FAM-ACC CCG CAT TAC GTT TGG TGG ACC-BHQ1-3’ |  |
| N2,  SARS-CoV-2 | F: 5’-TTA CAA ACA TTG GCC GCA AA-3’ | U.S. Centers for Disease Control and Prevention (CDC) 2020 |
|  | R: 5’-GCG CGA CAT TCC GAA GAA-3’ |  |
|  | Probe: 5’-FAM-ACA ATT TGC CCC CAG CGC TTC AG-BHQ1-3’ |  |
| M-Gene,  BCoV | F: 5’- CTGGAAGTTGGTGGAGTT – 3’ | Decaro et al. 2008 |
|  | R: 5’ – ATTATCGGCCTAACATACATC – 3’ |  |
|  | Probe: /56FAM/CCTTCATATCTATACACATCAAGTTGTT/3BHQ_1/ |  |

**Supplemental Table 2.** Specifications for qPCR reactions for N1, N2, and BCoV targets. All reactions prepared as 20 µL volumes. Reagents are shown in final concentration.

| **Reaction Component** | **N1,**  **SARS-CoV-2** | **N2,**  **SARS-CoV-2** | **M-Gene,**  **BCoV** |
| --- | --- | --- | --- |
| cDNA Template | 2 µL | 2 µL | 2 µL |
| 2X TaqMan Fast Advanced MasterMix | 1 U | 1 U | 1 U |
| Forward Primer | 0.5 µM | 0.5 µM | 0.6 µM |
| Reverse Primer | 0.5 µM | 0.5 µM | 0.6 µM |
| Probe | 0.13 µM | 0.13 µM | 0.2 µM |

**Supplemental Table 3.** Sequences of the synthetic DNA and RNA Ultramers used for the M-Gene target of the BCoV assay.

| Ultramer | Sequence | Length (bp) |
| --- | --- | --- |
| DNA Ultramer | 5’- CTGGAAGTTGGTGGAGTTT  CAACCCAGAAACAAACAACTTGATGTGTATAGATATGAAGGGAAGGATGTATGTTAGGCCGATAAT - 3’ | 85 |
| RNA Ultramer | 5’ - AUUAUCGGCCUAACAUACAUCCUUCCCUUCAUAUCUAUACACAUCAAGUUGUUUGUUUCUGGGUUGAAACUCCACCAACUUCCAG - 3’ | 85 |


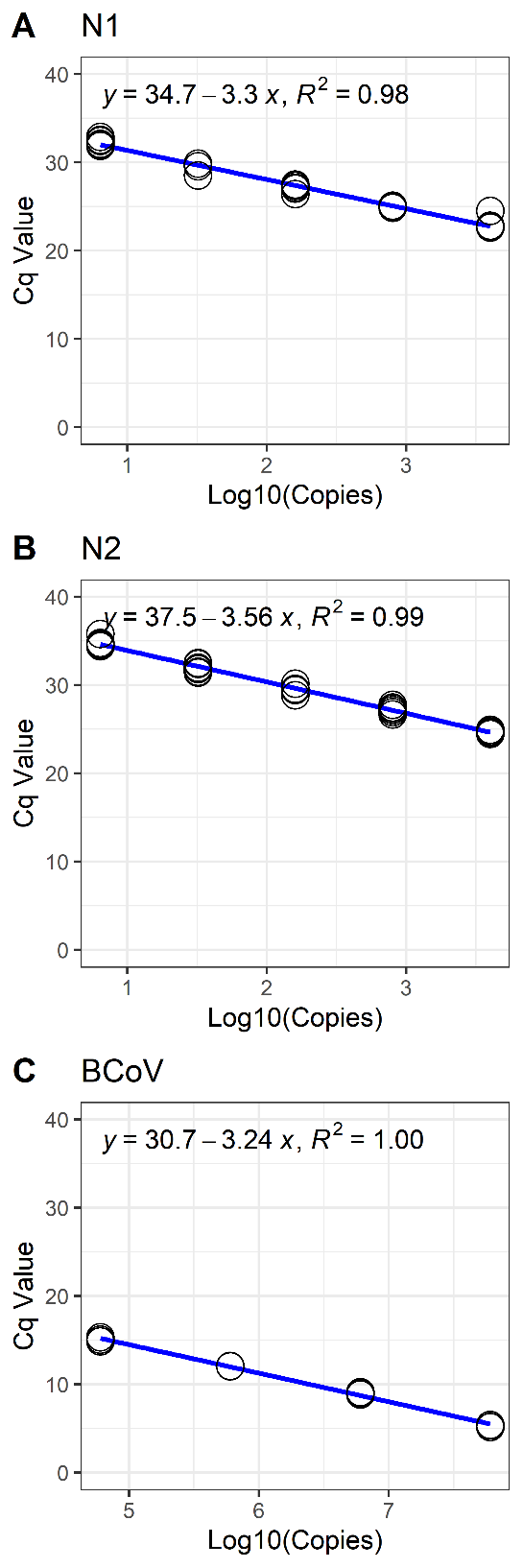

**Supplemental Figure 3.** Standard curves for the N1, N2, and BCoV two-step RT-qPCR assays. N1 and N2 standard curves were generated using a SARS-CoV-2 plasmid control, linearized by enzymatic digestion with ScaI-HF, and assayed in triplicate (N = 2). The BCoV curve was generated using a synthesized DNA Ultramer for the M-Gene of BCoV, and assayed in triplicate (N = 1).


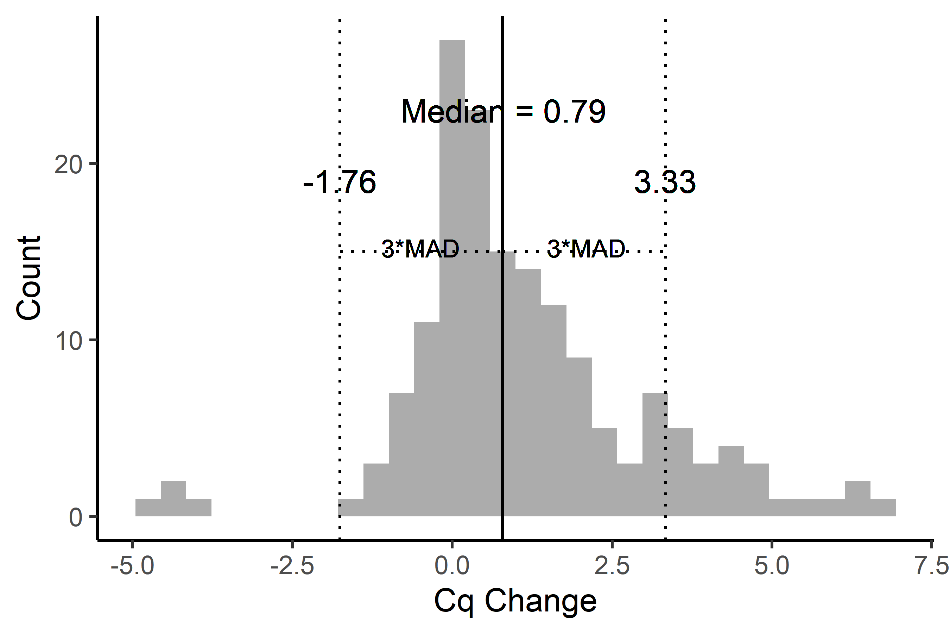


**Supplemental Figure 4.** Inhibition controls (N = 165). Change in Cq values between sample, spiked with an RNA Ultramer of BCoV target, and PCR-grade water, spiked with RNA Ultramer of BCoV target. The median Cq change was 0.76, but ranged from -4.64 to 6.85. Samples were considered inhibited if the Cq change between sample and control was greater than 3.33 cycles (equivalent to three times the median absolute deviation (3*MAD).


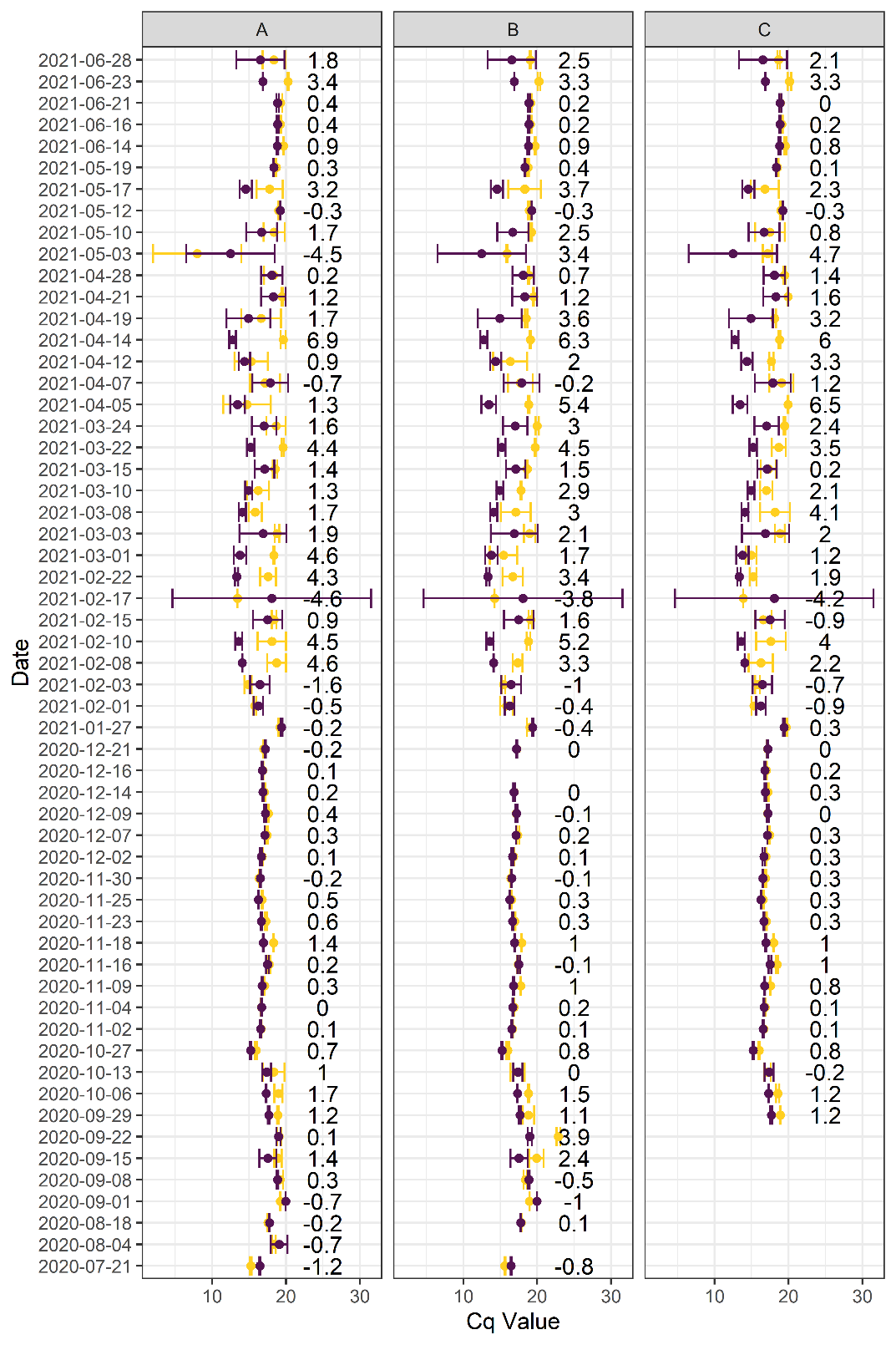


**Supplemental Figure 5.** Assessment of inhibition in RNA extracts from influent wastewater samples, organized by collection date and WRF (A, B, and C). Cq of sample (yellow), and control (purple), annotated with the change in Cq between the two. Error bars reflect the standard deviation of the three qPCR technical replicates.

**Supplemental Table 4.** Environmental Microbiology Minimum Information (EMMI) Guidelines (Borchardt et al. 2021).

| **EMMI Reporting Element** | **Location** |
| --- | --- |
| **ENVIRONMENTAL SAMPLING** |  |
| Sampling procedure, sample amount and number | Methods |
| Sampling locations and dates | Methods |
| Sample storage conditions and duration | Methods |
| Sampling negative control* | Methods |
| Sampling positive control* | Methods |
| **SAMPLE TREATMENT** |  |
| Treatment procedure and reagents | Methods |
| Treatment negative control* (e.g. filter eluent) | Methods |
| Treatment positive control* (may be combined with sampling positive control) | Methods |
| **SAMPLE REDUCTION** |  |
| Reduction procedure | NA |
| Concentration factor | NA |
| Reduction negative control* (may be combined with treatment negative control) | NA |
| Reduction positive control* (may be combined with sampling and treatment positive control) | NA |
| **NUCLEIC ACID EXTRACTION** |  |
| Extraction procedure | Methods |
| Concentration factor: amount extracted and amount obtained | NA |
| Extract storage conditions and duration | Methods |
| Extraction negative control | Methods |
| Extraction positive control | Methods |
| **REVERSE TRANSCRIPTION** |  |
| One or two step reaction | Methods |
| RT reaction temperatures and times | Methods |
| RT reaction reagents and concentrations | Methods |
| Priming method | Methods |
| Reaction volume, template amount added | Methods |
| cDNA storage conditions and duration | Methods |
| RT negative control | Methods |
| RT positive control | Methods |
| Inhibition assessment procedure | Methods |
| Number of samples tested and found inhibited | Results |
| Inhibition control description (if control used) | Methods |
| **qPCR** |  |
| Target gene name, amplicon length | Methods |
| Thermocycling temperatures and times | Methods |
| Master mix composition, vendors, concentrations | Methods |
| Additives, vendors, and concentrations | Methods |
| Template amount added, pre-treatment (if any) | Methods |
| Primer conc., vendor, sequence, reference | Methods, Supplemental |
| Hydrolysis probe concentration, dye and quencher, vendor, sequence, reference | Methods, Supplemental |
| Instrumentation | Methods |
| Amplicon confirmation method (e.g. probe, melt curve, gel, sequencing) | NA |
| Equivalent volume of sample analyzed by PCR | Discussion |
| qPCR negative control | Methods |
| qPCR positive control | Methods |
| Inhibition assessment procedure | Methods |
| Number of samples tested and found inhibited | Results |
| Inhibition control description (if control used) | Methods |
| **ANALYSIS - qPCR** |  |
| Accounted for negative controls that failed (e.g. Cq value separation between positives and sample contamination) | Results |
| Technical replicates: number, calculations, and summary statistics performed | Methods, Supplementary Data |
| Calibration standards, description and source | Methods |
| Standards quantification method | Methods |
| Calibration curve slope (i.e., PCR efficiency) | Supplementary Data, Supplemental Figure 3 |
| Calibration curve R^2 | Supplemental Figure 3 |
| Lowest standard measured of 95% LOD | NA |
| Cq value determination method (auto or manual threshold placement) | Methods |

**Supplemental Text 1.** Additional Details for Statistical Analyses.

**Recovery.** Normality of recovery rates was assessed using the Shapiro-Wilk test. Equality of recovery rates among the three WRFs was compared using the Kruskal–Wallis test followed by post hoc analysis by Dunn’s Multiple Comparison test with Bonferroni-Holm corrected p-values. The relationship between BCoV recovery, TSS, and influent flow was assessed with Spearman’s correlation.

**Detection of N1 and N2 Targets.** Pearson’s Chi Squared test was used to compare the frequency of detection between the N1 and N2 gene targets as well as the frequency of SARS-CoV-2 detection among the three WRFs.

**Cq Values.** Normality of observed Cq values was assessed using the Shapiro-Wilk test. Equality of Cq values among the N1 and N2 assays was assessed with the Mann-Whitney test.

**Viral Concentration.** Normality of viral concentration was assessed using the Shapiro-Wilk test. Equality of viral concentration was compared between the N1 and N2 targets using the Mann-Whitney test. The relationship between N1 and N2 viral concentrations was assessed with Spearman’s correlation. Equality of viral concentration was compared between WRFs using the Kruskall-Wallis test. The relationship between BCoV recovery, TSS, and influent flow was assessed with Spearman’s correlation.

**Viral Load.** Normality of viral load was assessed using the Shapiro-Wilk test. Equality of viral load was compared between the N1 and N2 targets using the Mann-Whitney test. The relationship between N1 and N2 viral load was assessed with Spearman’s correlation. Equality of viral concentration was compared between WRFs using the Kruskall-Wallis test followed by post hoc analysis by Dunn’s Multiple Comparison test with Bonferroni-Holm corrected p-values.


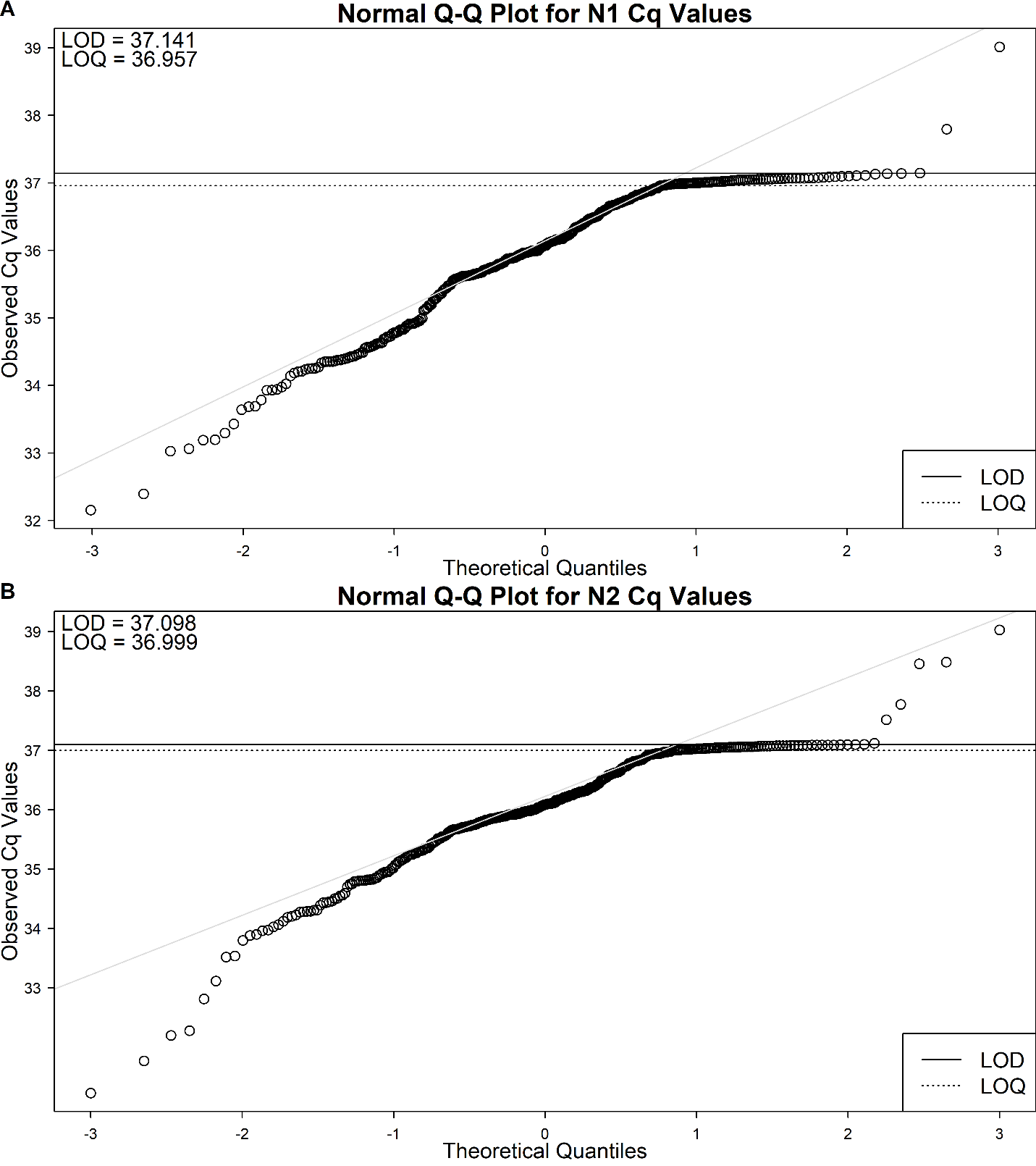

**Supplemental Figure 6.** Limits of Detection (LoD) and Quantification (LoQ) for the N1 and N2 assays. Observed Cq values for each assay plotted against normal quantile-quantile plots of the Cq values. Deviations from the y = x line were considered as artifacts of assay detections limits. The plotted sequences of points were scanned to identify the inflection points in the normal q-q plots (intersections with horizonal lines). The lesser value inflection point was used as an estimate for the limit of quantification and the greater value inflection point as an estimate for the limit of detection (beyond which observations were considered spurious and treated similarly to results of negative assays).

**Supplemental Table 5.** Summary of the number of RT-qPCR assay replicates and extraction replicates above the Limit of Quantification (LoQ), between the LoQ and Limit of Detection (LoD), and below the LoD.

| Facility | Target | Influent Samples | Extraction Replicates | Technical Replicates | Extraction Replicates with Assays Below LoD | Extraction Replicates with  Assays Between LoD and LoQ | Extraction Replicates Above LoQ | Technical Replicates No Amp | Technical Replicates Below LoD | Technical Replicates Between LoD and LoQ | Technical Replicates Above LoQ |
| --- | --- | --- | --- | --- | --- | --- | --- | --- | --- | --- | --- |
| A | N1 | 85 | 310 | 930 | 229/310 (74%) | 240/310 (77%) | 70/310 (23%) | 809/930 (87%) | 1/930 (0.1%) | 19/930 (2%) | 101/930 (11%) |
| A | N2 | 83 | 303 | 926 | 217/303 (72%) | 230/303 (76%) | 73/303 (24%) | 804/926 (87%) | 1/926 (0.1%) | 21/926 (2%) | 100/926 (11%) |
| B | N1 | 85 | 309 | 927 | 207/309 (67%) | 230/309 (74%) | 79/309 (26%) | 781/927 (84%) | 0/927 (0%) | 40/927 (4%) | 106/927 (11%) |
| B | N2 | 83 | 303 | 927 | 197/303 (65%) | 219/303 (72%) | 84/303 (28%) | 777/927 (84%) | 3/927 (0.3%) | 32/927 (3%) | 115/927 (12%) |
| C | N1 | 84 | 304 | 912 | 219/304 (72%) | 235/304 (69%) | 69/304 (23%) | 796/912 (87%) | 2/912 (0.2%) | 23/912 (3%) | 91/912 (10%) |
| C | N2 | 82 | 295 | 902 | 223/295 (76%) | 234/295 (79%) | 61/295 (21%) | 804/902 (89%) | 2/902 (0.2%) | 15/902 (2%) | 81/901 (9%) |


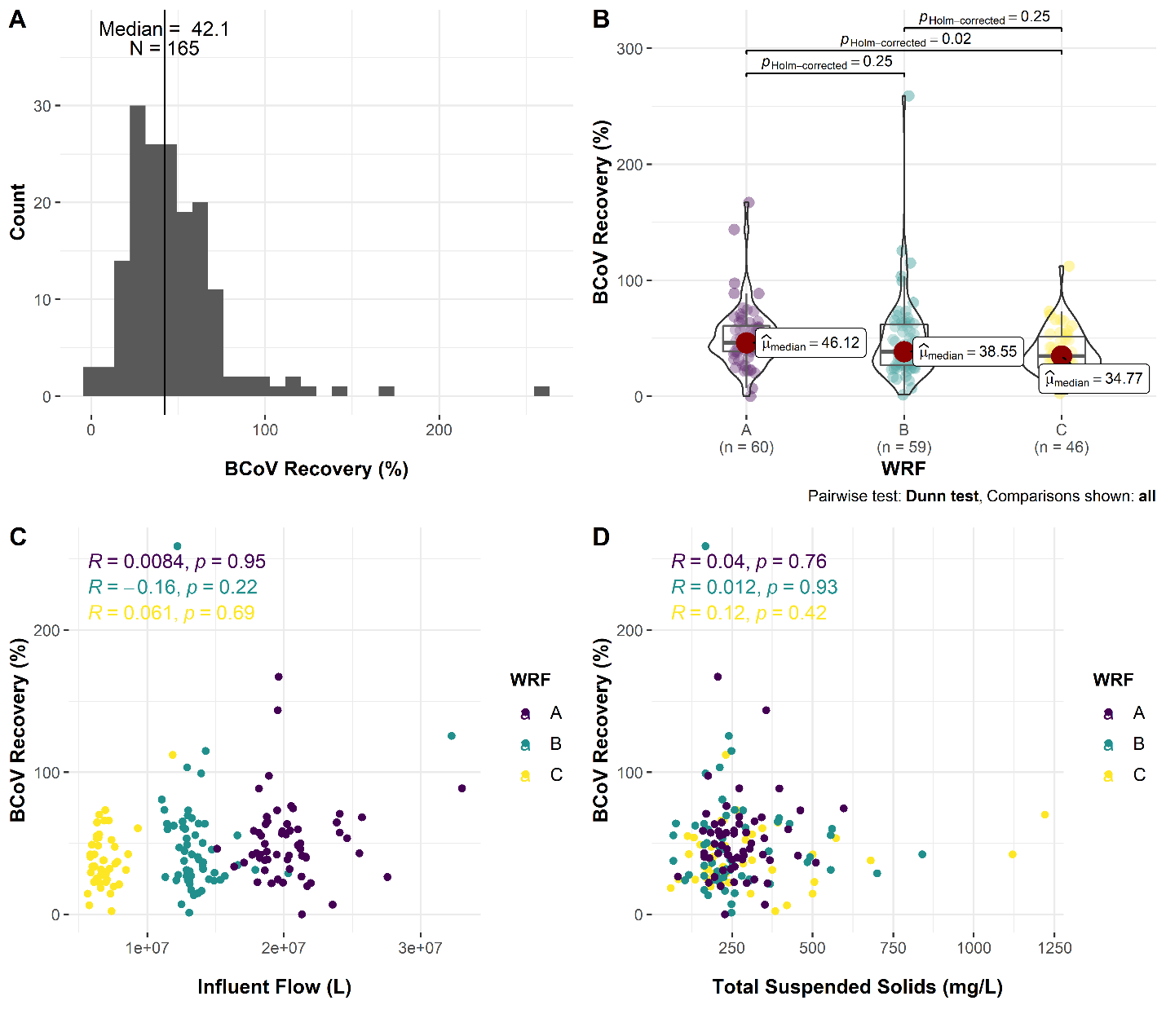


**Supplemental Figure 7.** Process controls for the recovery of bovine coronavirus from wastewater (BCoV). (A) Distribution of viral recovery of BCoV from 165 process controls. (B) Viral recovery assessed for influent samples, by source WRF. (C) Spearman’s correlation between viral recovery and influent flow. (D) Spearman’s correlation between viral recovery and total suspended solids.


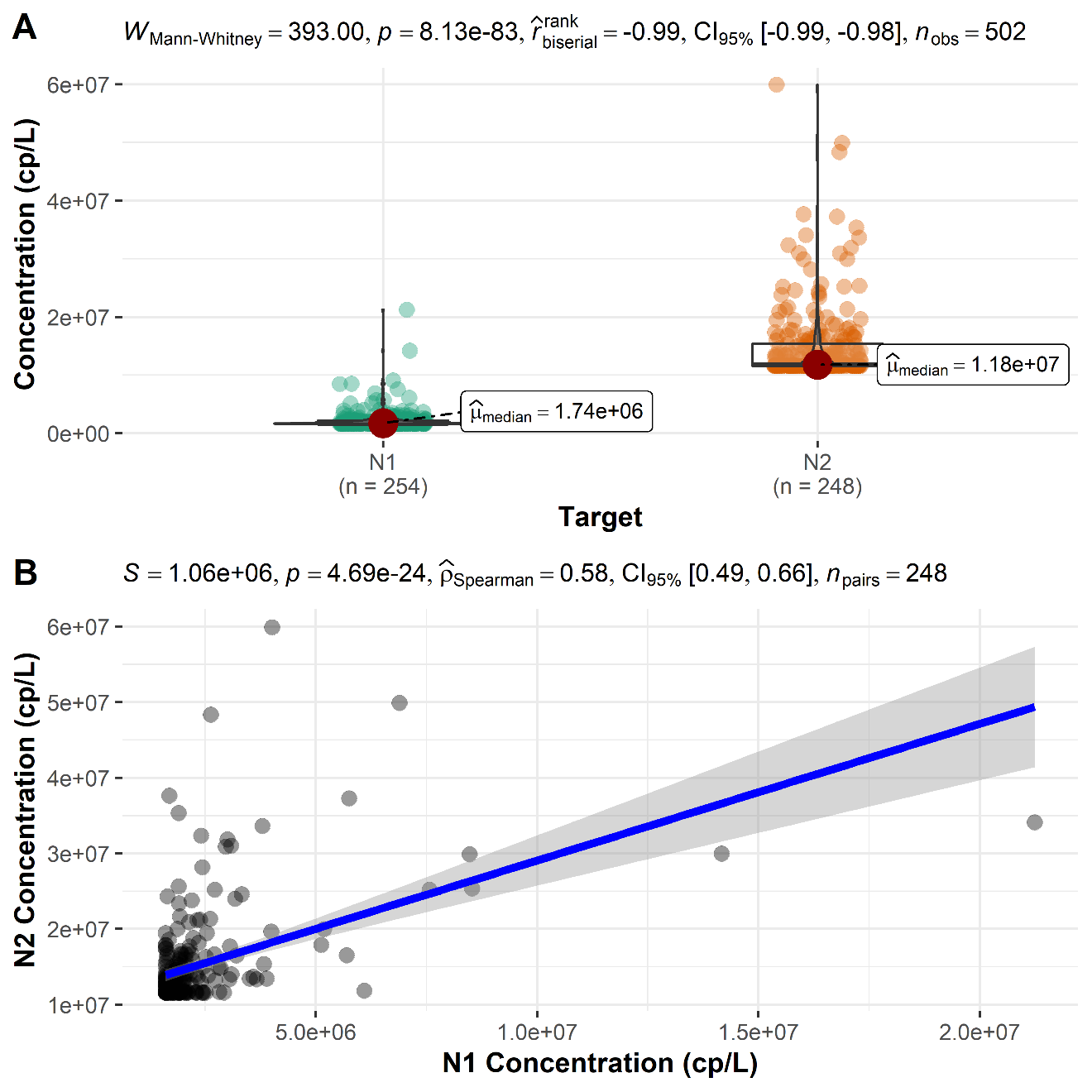


**Supplemental Figure 8.** Comparison of the N1 and N2 assays for quantification of SARS-CoV-2 concentration in wastewater. (A) Viral concentration of SARS-CoV-2 by the N1 and N2 assays. (B) Spearman’s correlation between the viral concentrations of SARS-CoV-2 determined by the N1 and N2 assays.


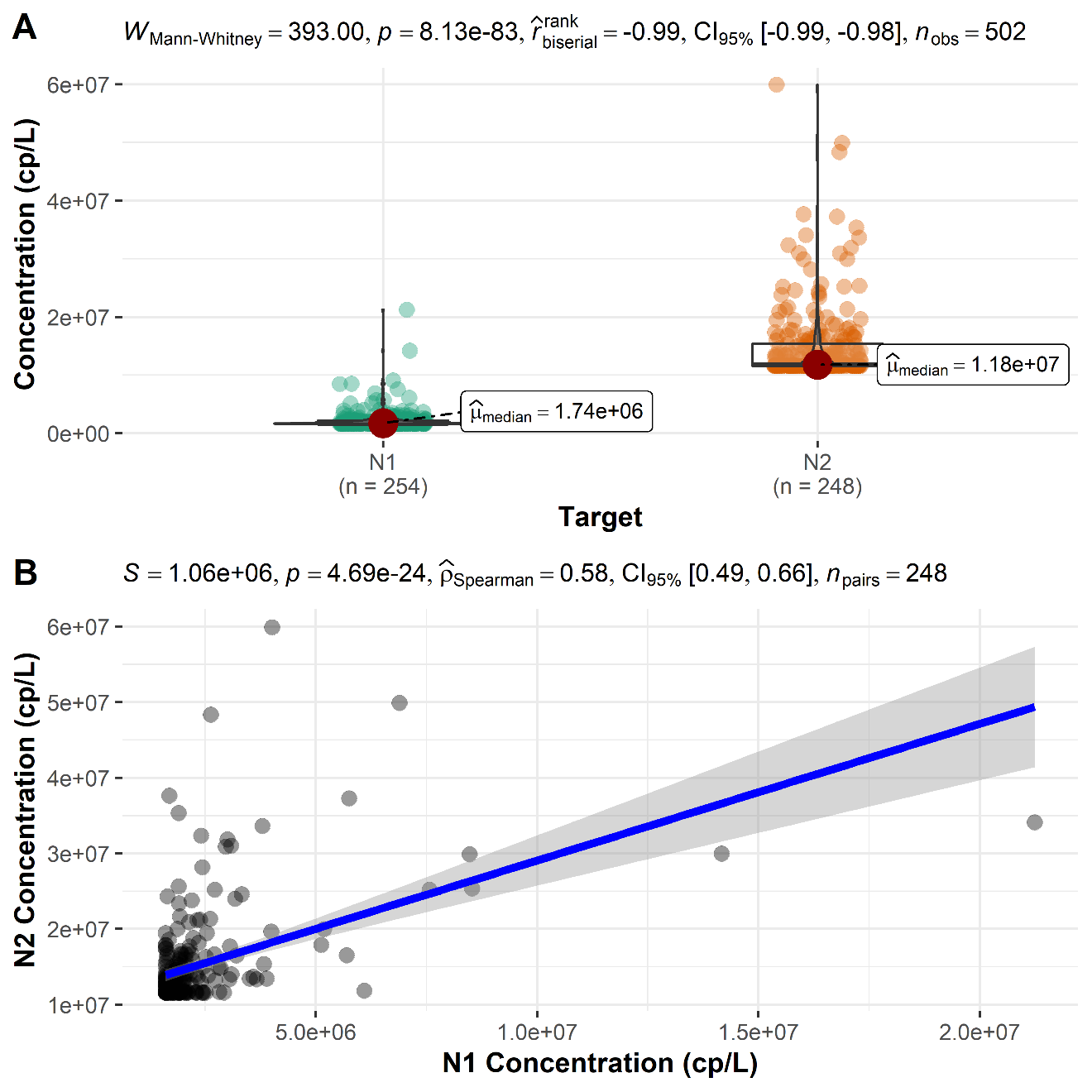


**Supplemental Figure 9.** Comparisons of the N1 and N2 assays to estimate daily viral load of SARS-CoV-2 in wastewater. (A) Daily viral load of SARS-CoV-2, determined by the N1 and N2 assays. (B) Spearman’s correlation between the daily viral load of SARS-CoV-2 determined by the N1 and N2 assays.


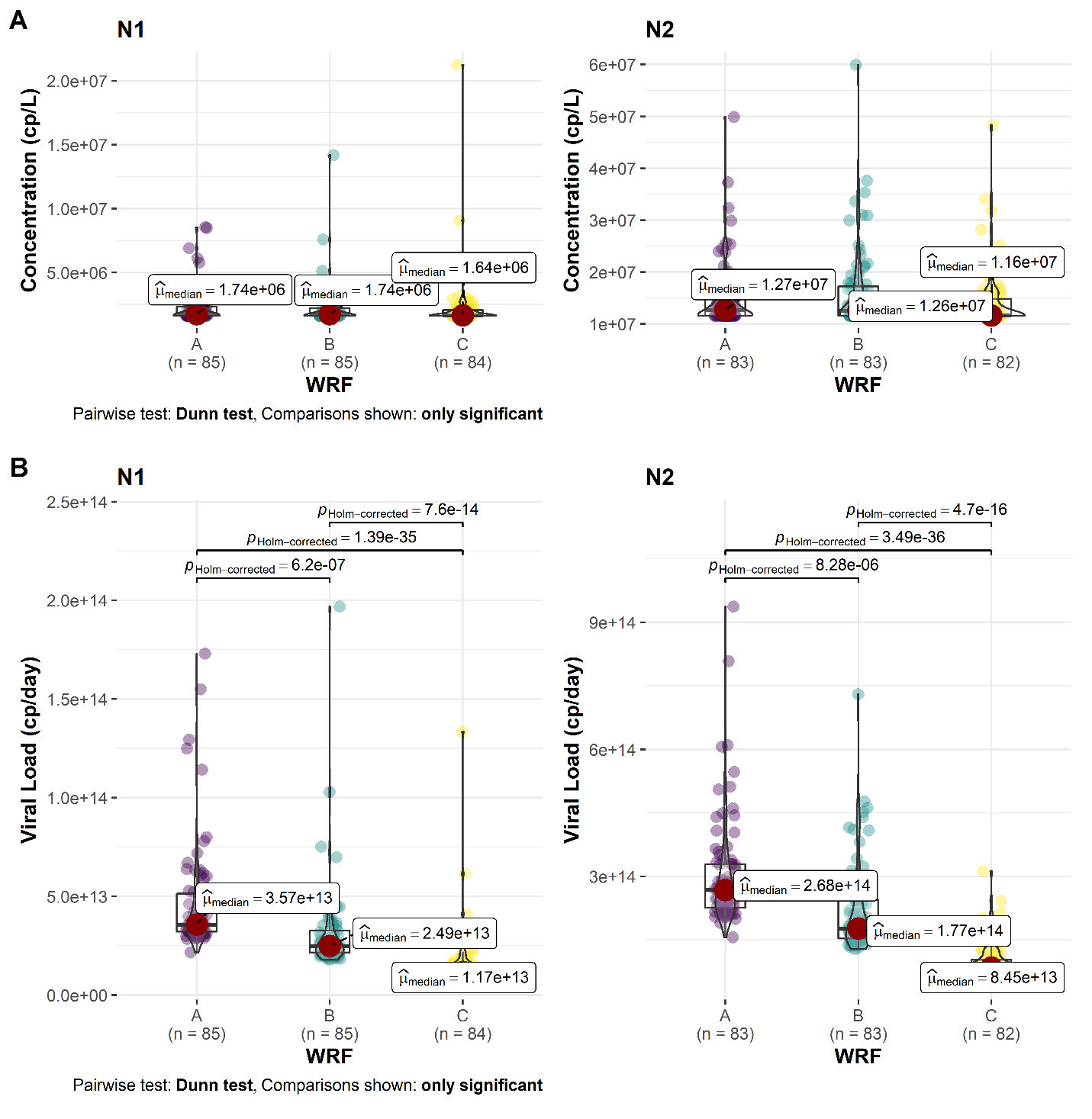


**Supplemental Figure 10.** Comparison of SARS-CoV-2 viral RNA quantified from three wastewater reclamation facilities. (A) Concentration of SARS-CoV-2 viral RNA in influent wastewater samples collected from WRF A, WRF B, and WRF C, by the N1 and N2 assays. (B) Daily viral load of SARS-CoV-2 viral RNA quantified from influent wastewater samples from WRF A, WRF B, and WRF C, by the N1 and N2 assays.


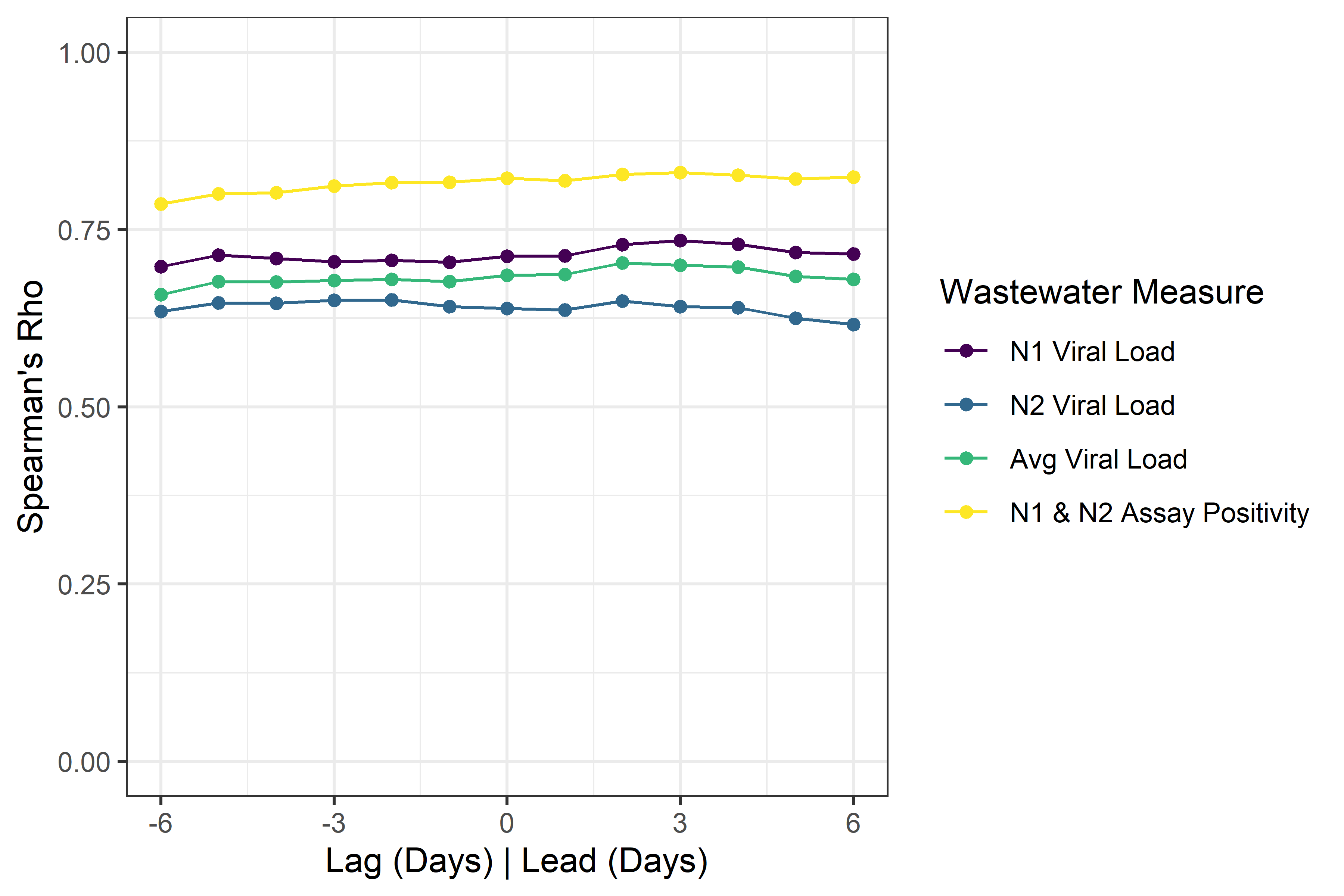


**Supplemental Figure 11.** Lead and lag times for wastewater surveillance of SARS-CoV-2 in Athens-Clarke County. Spearman’s Rho are reported for the correlation between measures of SARS-CoV-2 in wastewater (N1 viral load, N2 viral load, geometric mean of N1 and N2 viral loads “Avg Viral Load”, and total assay positivity) and reported cases of COVID-19 (per-capita, 7-dma). Correlations were assessed between wastewater surveillance and the number of reported cases six days prior (-6,0), and six days following (0,6) a given wastewater sample collection. All correlations presented are statistically significant (p < 0.001).
