# Supplementary material for: Direct wastewater extraction as a simple and effective method for SARS-CoV-2 surveillance and COVID-19 community-level monitoring
Source: FEMS Microbes. 2023 Jan 12;4:xtad004. doi: 10.1093/femsmc/xtad004 (PMC10117872; doi:10.1093/femsmc/xtad004)
Supplement: xtad004_Supplemental_Files [file xtad004_supplemental_files.zip › FEMSMC-2022-022 reviews and author response.docx]

**Reviews of manuscript FEMSMC-2022-022**

**Reviewer 1**

**Recommendation: Minor Revision**

This study measured N1 and N2 genes in wastewater by a direct extraction method with no concentration. The results demonstrated good performance and correlation with Covid-19 cases. The study was well planned and included all necessary controls and performance testing. The introduction and discussion are well presented and organized. The necessary results are included in figures and tables. There is one suggestion for the determination of LOD and LOQ, if the authors could explain more on the rationale for using the current method instead of a common method of using standards and lowering their concentrations. Moreover, in the abstract it would be helpful to include the LOD LOQ values when determining the positive percentage. And to mention the type of spike (BCoV) when mentioning recovery values.

**Reviewer 2 - Maria Inês Z. Sato**

**Recommendation: Minor Revision**

The present manuscript reports the implementation and evaluation of a simplified workflow for SARS-CoV-2 from wastewater (skipping the pre-concentration step), using a direct column-based extraction. In the literature there are substantial publications evaluating different sample concentration methods for different laboratory conditions with great differences in virus recovery and cost. The purpose of the paper is to provide a simple, fast, cheap and effective method for SARS-CoV-2 surveillance in wastewaters.

The manuscript is within the scope of the Journal, presenting current methodological advances that are of interest to global wastewater monitoring programs. It is well structured, meeting the technical and scientific requirements of the Journal. The abstract summarizes the main points of the research with a comprehensive overview of the entire study. The introduction provides adequate context and background of the main topics covered in the study and clearly states the purpose of the work. The experimental design is well structured and the methods and statistical analysis are presented in detail in the manuscript and supplementary material. The results are clearly reported, treated statistically and discussed in the light of scientific knowledge, exploring the significance of the data. The conclusions are appropriated and achieved the proposed objectives.

A limitation of the present study is the high detection and quantifications limits of direct extraction procedure. Although the authors has discussed properly these aspects and, the linear regression between reported cases and wastewater assay positivity estimated that SARS-CoV-2 viral RNA could be positively detected in wastewater when cases of COVID-19 exceed approximately five cases per day, per 100,000 individuals, this limitation should be noted in the abstract and conclusions. Data analysis considered the SARS-CoV-2 concentrations bellow LoD and LoQ, but it was unclear what percentage of results fell within these ranges. Figure 2 displays the values below LoD but not the values between LoD-LoQ. Furthermore, figure 2 is not easy to visualize, as N1 load values and the geometric mean of N1+N2 are flattened, as they are one log lower than the N2 values.

In conclusion, the manuscript is relevant and we recommend the publication with minor revision, reinforcing the limitation of the method in relation the LoD and LoQ, especially for the non-epidemic/pandemic scenario.

**Author's Response to Decision Letter for (FEMSMC-2022-022)**

Author's Response to Reviewers:

Reviewer: 1

Response: Thank you for your comment! We appreciate the need for transparent and accurate reporting of the limits of detection and quantification in RT-qPCR and hope to provide that clarity here. Rather than determining the LoD and LoQ from our analytical standards, we determined the LoQ and LoD by evaluating the Cq values of our sample assays. This approach has several advantages. By examining empirical Cq values, our estimates of the LoD and LoQ reflect variability and error associated with the analytical process, rather than errors associated with the preparation of analytical standards. Our approach was more cost-effective and informative than examining the Cq values from our standard curves. With our empirical Cq values, we examined over 2,750 RT-qPCR replicate assays for each target. This sampling effort is the equivalent to running over 180 5-point standard curves for each target, in triplicate. The Cq values from the empirical assays yielded a dataset of continuous Cq values, rather than the discrete values obtained from the standard curves. Moreover, many of these replicates approximated the lower limits of detection for the assay. We assert that with this method, we are able to estimate the LoD and LoQ more precisely than with the use of the standard curves alone.

We determined the limit of detection (LoD) and limit of quantification (LoQ) by referring to the most basic definitions of these measures as provided by the Clinical and Laboratory Standards Institute, where the LoD is defined as the smallest amount of analyte that can be reliably detected by the method, and the LoQ is defined as the smallest amount of analyte can reliably quantified by the method (The Clinical and Laboratory Standards Institute 2012). For RT-qPCR, the Cq results are usually normally distributed (Forootan et al. 2017); thus, we defined the limit of quantification and detection based on the deviations of observed Cq values from the expected normal distribution.

In our dataset, we observed that empirical Cq values for the N1 assay measured between Cq 32 and Cq 37 were approximately normally distributed. We observed a distinct inflection point at 36.96. Beyond this value, we could not expect to reliably measure the target analyte using the standard curve. We defined this inflection point as the LoQ. There were 84 assays that amplified after the LoQ, but another inflection point was observed at Cq 37.14. Cq values beyond this inflection point could not be reliably detected or distinguished from noise. We defined this Cq value as the LoD.

The LoD and LoQ for the N2 assay were determined through the same means. Empirical Cq values for the N2 assay measured between 31 and 37 were normally distributed. We observed a distinct inflection point at Cq 37. Beyond this value, we could not expect to reliably measure the target analyte using the standard curve. We defined this inflection point as the LoQ. There were 74 assays that amplified beyond the LoQ which were consistently detected between Cq 37.10. Cq values beyond 37.10 this inflection point could not be reliably detected or distinguished from noise. We defined this Cq value as the Limit of Detection.

The justification and methodology for the LoD and LoQ determination have been expanded upon within the Methods section of the manuscript. The LoQ value has been added the abstract.

The mention of viral recovery has now been clarified throughout the manuscript to specify BCoV recovery. Thank you!

Reviewer 2: Response

Thank you for your thoughtful review of this work! The abstract and conclusions has been updated to note the high limit of detection of the method, but the applicability of this approach even during periods of low incidence. Figure 2 has been updated to improve the visualization of the overall trends for the N1, N2, and N1+N2 viral loads. Additionally, we have included a detailed summary of the number of RT-qPCR and extraction replicates below and between each detection limit in Supplemental Table 5. Thank you! We appreciate your comments and recommendations.

**Reviews of manuscript FEMSMC-2022-022.r1**

**Reviewer 1**

**Recommendation: Accept**

The manuscript has been improved and now suitable for publication.

**Reviewer 2 - Maria Inês Z. Sato**

**Recommendation: Accept**

I appreciated that the authors considered my notes and suggestions in reviewing the manuscript.
